# Identification and validation of genes associated with prognosis of cisplatin-resistant ovarian cancer

**DOI:** 10.1186/s12885-024-12264-z

**Published:** 2024-08-05

**Authors:** Dajiang Liu, Ruiyun Li, Yidan Wang, Dan Li, Leilei Li

**Affiliations:** 1 Department of Obstetrics and Gynecology, The First Hospital of Lanzhou University, Lanzhou, China; 2 The First Clinical Medical College, Lanzhou University, Lanzhou, China

**Keywords:** Ovarian cancer, Cisplatin-resistant, Bioinformation, Risk score model

## Abstract

**Purpose:**

To investigate the role of prognostic genes related to cisplatin resistance in ovarian cancer during disease progression.

**Method:**

The gene expression profile of the NCI-60 cell line was acquired through comprehensive analysis of the GEO database accession GSE116439. We performed a thorough analysis of gene expression differences in samples from seven individuals exposed to cisplatin concentrations of 0 nM compared to seven samples exposed to 15000 nM over a 24-h period. Key genes were initially identified through LASSO regression, followed by their enrichment through differential gene function analysis (GO) and pathway enrichment analysis (KEGG). Subsequently, a prognostic risk model was established for these key genes. The prognostic model's performance was assessed through K-M survival curves and ROC curves. To examine the variance in immune cell infiltration between the high and low-risk groups, CIBERSORTx analysis was employed. Finally, validation of prognostic gene expression in cisplatin-resistant ovarian cancer was carried out using clinical samples, employing RT-qPCR and Western Blot techniques.

**Results:**

A total of 132 differential genes were found between cisplatin resistance and control group, and 8 key prognostic genes were selected by analysis, namely VPS13B, PLGRKT, CDKAL1, TBC1D22A, TAP1, PPP3CA, CUX1 and PPP1R15A. The efficacy of the risk assessment model derived from prognostic biomarkers, as indicated by favorable performance on both Kaplan–Meier survival curves and ROC curves. Significant variations in the abundance of Macrophages M1, T cells CD4 memory resting, T cells follicular helper, and T cells gamma delta were observed between the high and low-risk groups. To further validate our findings, RT-qPCR and Western Blot analyses were employed, confirming differential expression of the identified eight key genes between the two groups.

**Conclusion:**

VPS13B, TBC1D22A, PPP3CA, CUX1 and PPP1R15A were identified as poor prognostic genes of cisplatin resistance in ovarian cancer, while PLGRKT, CDKAL1 and TAP1 were identified as good prognostic genes. This offers a novel perspective for future advancements in ovarian cancer treatment, suggesting potential avenues for the development of new therapeutic targets.

**Supplementary Information:**

The online version contains supplementary material available at 10.1186/s12885-024-12264-z.

## Background

Ovarian cancer (OC) is a highly lethal malignancy in women and continues to pose a significant global public health challenge. According to epidemiological data spanning from 1990 to 2019, the worldwide incidence of OC was approximately 294.42 × 10^3^ cases, with an associated mortality of about 198.41 × 10^3^ cases. Notably, China accounted for around 45.48 × 10^3^ incident cases and approximately 29.09 × 10^3^ deaths due to this disease during the same period. In the year of analysis (2019), there was an alarming rise in both standardized death rate (2.88 per 100,000) and crude death rate (4.17 per 100,000) attributed to ovarian cancer [1, 2]. The elevated mortality rate of ovarian cancer is linked to its subtle and atypical early symptoms, including abdominal bloating, pelvic pain, early satiety, and changes in bowel function. These manifestations often lead to diagnostic challenges and confusion with other medical conditions [3, 4]. Moreover, the early stages of the disease are characterized by a dearth of efficacious diagnostic methods. Furthermore, as the cancer progresses, malignant cells disseminate via hematogenous and lymphatic routes to various sites within the abdominal cavity including but not limited to the liver, lungs, brain. Consequently, delayed diagnosis significantly compromises prognosis for OC treatment [4]. Currently, the management of OC primarily relies on surgical resection and adjuvant chemotherapy. Surgical resection aims to maximize tumor tissue removal, however, due to the invasive nature of abdominal and pelvic cavity operations, it poses challenges in terms of difficulty, slow postoperative recovery, and potential complications [5, 6]. Despite the sensitivity of OC to chemotherapy, the majority of patients experience relapse and rapid mortality. The resistance to chemotherapy and the challenges in disease management with current therapeutic approaches contribute to this phenomenon [4, 7].

OC is categorized into three primary types: epithelial, germ cell, and interstitial tumors. Within the epithelial type, there are five subtypes, namely low-grade serous cancer, high-grade serous cancer, endometrioid cancer, clear cell cancer, and mucinous cancer. Notably, high-grade serous ovarian cancer stands out as the most aggressive and deadliest subtype [4, 8]. If ovarian cancer is treated and diagnosed early, the 5-year survival rate ranges from 80 to 90 percent when it remains localized within the ovaries; however, this rate drops to less than 30 percent when there is infiltration of adjacent pelvic structures or distant organ metastasis [4, 9]. Cisplatin exerts a significant impact on the initial treatment of ovarian cancer by inducing cross-linking and RNA destruction, effectively eradicating proliferating cancer cells. Failure to promptly repair damaged DNA triggers the DNA damage response, leading to apoptosis activation [10]. However, the majority of patients eventually develop resistance to cisplatin following repeated exposure, thereby resulting in tumor recurrence. The acquisition of cisplatin resistance is an intricate process, involving multiple mechanisms[11]. Ziliang Wang and colleagues have shown a noteworthy increase in the expression of fibrillin-1 in ovarian cancer tissues. This upregulation subsequently triggers the downstream pathway through vascular endothelial growth factor receptor 2. Ultimately, this leads to altered gene expression related to angiogenesis and glycolysis mediated by transcription factor 2, thereby promoting cisplatin resistance [12]. Sipei Nie et al. reported an upregulation of ALKBH5 in cisplatin-resistant ovarian epithelial carcinoma, where it forms a loop with HOXA10 to facilitate the development of cisplatin resistance in cancer cells [13]. Chemotherapy resistance constitutes the primary cause of treatment failure in ovarian cancer, necessitating urgent investigation into the underlying mechanisms and identification of novel therapeutic targets.

This study involved a thorough analysis of biological information to identify 132 genes linked to the onset of cisplatin resistance in OC. Subsequently, LASSO regression analysis identified 8 key genes. To validate their expression, clinical samples were collected and analyzed using RT-qPCR and Western Blot techniques. VPS13B, TBC1D22A, PPP3CA, CUX1 and PPP1R15A were identified as poor prognostic genes for cisplatin resistance in ovarian cancer, while PLGRKT, CDKAL1 and TAP1 were identified as good prognostic genes. These associated prognostic signature genes can potentially facilitate early detection and improved treatment of ovarian cancer, providing novel insights into the clinical diagnosis and management of this disease.

## Materials and methods

### Data download

The GSE116439 dataset, obtained through the R package GEOquery from the GEO database, encompasses a gene expression profile derived from the NCI-60 cell line exposed to cisplatin, an anticancer drug. We screened 14 samples from GSE116439 for subsequent analysis, comprising of 7 control samples treated with 0 nM cisplatin for a duration of 24 h and 7 experimental samples treated with a concentration of 15000 nM cisplatin for the same time period. The GSE116439 dataset is based on the GPL571 [HG-U133A_2] Affymetrix Human Genome U133A 2.0 Array platform, and the probe annotation of the dataset is conducted using the chip GPL platform file.

We retrieved the gene expression profile data and survival data of ovarian cancer patients from the TCGA database for subsequent bioinformatics analysis. Additionally, we obtained the maf file for mutation analysis and excluded samples with missing survival data in ovarian cancer, resulting in a final dataset comprising 373 tumor tissue samples, no normal tissue samples were available.

### Cisplatin drug-related differentially expressed genes

To discern alterations in gene expression subsequent to cisplatin exposure, we conducted a differential analysis using the limma packages, aiming to identify differentially expressed genes (DEGs) between the control and experimental groups. The criteria for selecting DEGs for further exploration were set at |logFC|> 1 and a P-value < 0.05. Genes meeting the criteria of logFC > 1 and *P*-value < 0.05 were categorized as up-regulated, while genes contrary to the criteria were classified as down-regulated. The outcomes of the differential analysis were visually presented through a volcano plot generated with the R package ggplot2, a heatmap created using the R package pheatmap, and a box plot constructed with the R package ggpubr.

### Differential gene function and pathway enrichment analysis

GO analysis is a widely employed approach for conducting comprehensive functional enrichment studies, encompassing biological processes (BP), molecular functions (MF), and cellular components (CC). KEGG stands as a widely employed repository for the comprehensive storage of genomic data, biological pathways, disease information, and pharmaceutical compounds. GO and KEGG enrichment analyses on DEGs associated with cisplatin were carried out using the R-package clusterProfiler. The selection criteria included a *P*-value < 0.05 and a false discovery rate (FDR or q value) < 0.05.

### GSEA enrichment and GSVA analysis

GSEA evaluates the distribution trend of genes in a pre-defined gene set within a phenotypically ranked gene list to assess their contribution to the phenotype. In this study, differentially expressed genes were divided into high and low phenotype relevance groups. The clusterProfiler package enriched all DEGs in these two groups. GSVA, a non-parametric unsupervised algorithm, was then applied using the R language GSVA (version 1.42.0) package. This transformed gene expression data from a matrix with a single gene as a feature to a matrix with a specific gene set as a feature. Each gene set underwent rank statistics, akin to the K-S test, resulting in an Enrichment Score (ES) matrix. This facilitated GSVA enrichment score determination for each sample and subsequent statistical analysis.

### Construct prognosis model based on TCGA data

We identified 132 genes with differential expression between the control and experimental groups, considered potential candidates. To assess their prognostic significance in OC, tumor samples were randomly split into a 3:2 ratio, with three as the training set and two as the validation set. Univariate Cox regression analysis was applied to the training set to identify genes significantly associated with survival (*P* value < 0.05). A prognostic model was constructed using Least Absolute Shrinkage and Selection Operator (LASSO) regression, incorporating only genes with non-zero regression coefficients. The Risk Score model yielded the risk score for each tumor sample, calculated as follows: Coef (genei) represents LASSO regression coefficient, expression (genei) denotes the gene's expression value, and n is the number of genes in the model.

$${\mathrm{riskScore}=\sum\limits_{\mathrm i}^{\mathrm n}\mathrm{Coef}\left({\mathrm{gene}}_{\mathrm i}\right)\ast\mathrm{Expression}\;}\left({\mathrm{gene}}_{\mathrm i}\right)$$

### Evaluation of prognostic models

The R-package survminer's surv_cutpoint function was utilized to determine the optimal cutoff value for distinguishing high and low-risk groups in the training set. Following this, Kaplan–Meier survival curve analysis and time-dependent ROC analysis were performed to evaluate the predictive accuracy of the model.

### Build a forecast nomogram

A nomogram, based on multiple regression analysis, utilizes a specific scale to assign scores, representing various variables within the multiple regression model. Ultimately, a total score is computed to predict the probability of event occurrence. We integrated the clinical features of ovarian cancer samples to identify the clinical characteristics significantly associated with survival. Following the Cox regression analysis results, we constructed a nomogram using the R package "rms."

### Immunoinfiltration analysis

We utilized the CIBERSORTx online tools (https://cibersortx.stanford.edu/) to evaluate immune cell infiltration in TCGA—OV data, obtaining abundance values for 22 distinct subtypes of immune cells. We utilized bar charts to visually represent the proportions of anticipated cells, employed Pearson correlation heat maps to illustrate the interrelationships among immune cells, and employed box plots to examine disparities in immune cell populations between high and low risk cohorts.

### Drug sensitivity prediction

Using the Cancer Genome Project (CGP) database, Ridge regression was applied to estimate the half-maximum inhibitory concentration (IC50) for each patient, and the prediction accuracy was evaluated through tenfold cross-validation. Significance in drug sensitivity differences between high and low-risk groups was determined by comparing *P* values, where *P* < 0.001 was considered statistically significant.

### Mutation analysis

Tumor mutation burden (TMB) quantifies the number of somatic nonsynonymous mutations in a specific genomic region. It typically represents the cumulative count of coding errors, base substitutions, and gene insertion or deletion errors detected per million bases. We employed Maftools (version 2.10.0), an R package specifically designed for TMB analysis. This allowed us to quantify somatic non-synonymous point mutations in each sample and assess the mutation frequency of genes in both high-risk and low-risk groups. Furthermore, we visually depicted these findings using an oncoplot waterfall plot.

### Chromosome localization analysis of prognostic genes

We will prepare the chromosome localization data, load the RCircos package in the R language environment, and import the data. Subsequently, we will utilize the functions within the RCircos package to generate a circular chromosome map and incorporate chromosomal location markers. The objective of interpreting the results of chromosome localization analysis is to discuss the distribution pattern of prognostic genes on chromosomes and explore the biological significance of these genes.

### ceRNA network analysis

The interacting microRNAs associated with genes significantly linked to prognosis were queried in the miRDB database (http://www.mirdb.org/), utilizing a Target Score > 88 as a filter. Subsequently, a query was performed in the starBase database to identify interactions of miRNAs (http://starbase.sysu.edu.cn/index.php). The intersection of these two database queries identified gene-miRNA interactions that were strongly associated with prognostic outcomes. The starBase database was queried to identify lncRNAs that interact with the aforementioned miRNAs, using a filtering condition of clipExpNum > 10. Finally, the ceRNA network diagram was constructed using Cytoscape based on the aforementioned query results.

### Quantitative real-time PCR

RNA extraction from tissues was performed using Trizol reagent, reverse transcribed into cDNA utilizing SweScript One-Step RT-PCR Kit, and subsequently amplified with appropriate primers (refer to Table S1) for validation of prognostic gene expression in the cisplatin-resistant group of ovarian cancer.

### Western Blot

Tissues were lysed with RIPA lysis buffer, and protein concentration was determined using the BCA protein assay kit. The protein was then separated on an SDS-PAGE gel and transferred to a PVDF membrane. Subsequently, the expression of the target protein was detected using the FUSION FX5 imaging system (Bio-Rad, Hercules, CA, USA) following continuous incubation with primary and secondary antibodies. The primary antibodies targeting the following proteins were used VPS13B (Abcam, ab139814, 1:1000), PLGRKT (Abcam, ab169531, 1:1000), CDKAL1 (Abcam, ab237525, 1:1000), TBC1D22A (Abcam, ab234723, 1:1000), TAP1 (Abcam, ab314745, 1:1000), PPP3CA (Abcam, ab265130, 1:1000), CUX1 (Abclonal, A2213, 1:1000), and PPP1R15A (Abclonal, A16260, 1:1000). The secondary antibody used was β-actin (Abclonal, AC026, 1:10,000).

### Statistical analysis

Data processing and analysis in this study used R software (Version 4.1.2), presenting continuous variables as mean ± standard deviation. The Wilcoxon rank sum test (Mann–Whitney U test) compared two groups, while the Kruskal–Wallis test assessed three or more groups. Chi-square tests or Fisher exact tests determined statistical significance for comparing and analyzing two sets of categorical variables. Unless specified otherwise, Spearman correlation analysis calculated correlation coefficients between different molecules, with a significance threshold at *P* < 0.05.

## Results

### Analysis of cisplatin drug-related gene differences

**Fig. 1:**
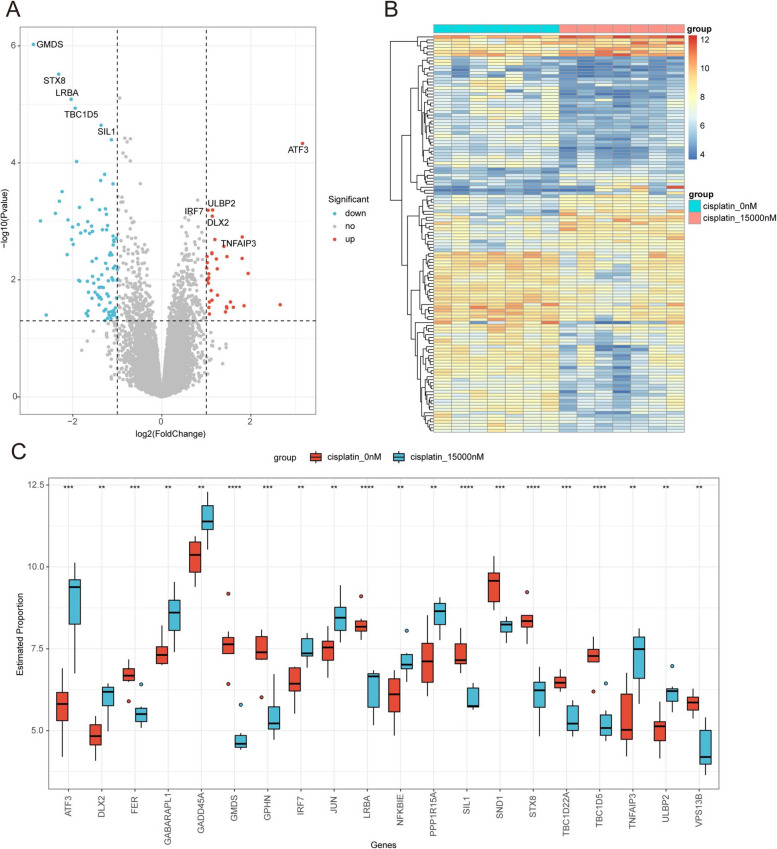
Differential expression analysis of cisplatin drug-related genes. **A** Volcano plot of the DEGs. **B** Heat map depicting the distribution of DEGs. **C** Box plots displaying the expression distribution of the top 10 up-regulated and down-regulated genes in the experimental and control groups

The differential gene expression analysis was conducted between the cell samples treated with cisplatin at a concentration of 15000 nM and those untreated for 24 h. A total of 132 genes exhibiting significant differences in expression were identified (Table S2), including 35 up-regulated genes and 97 down-regulated genes. Differential gene expression analysis results are illustrated in the volcano plot (Fig. 1A). The expression distribution of 132 significantly differentially expressed genes in experimental and control samples is shown in heat maps (Fig. 1B). Additionally, a boxplot was created to visualize expression differences between the experimental and control groups for 20 significant variant genes (Fig. 1C).

### GO and KEGG analysis of cisplatin drug-related DEGs

**Fig. 2:**
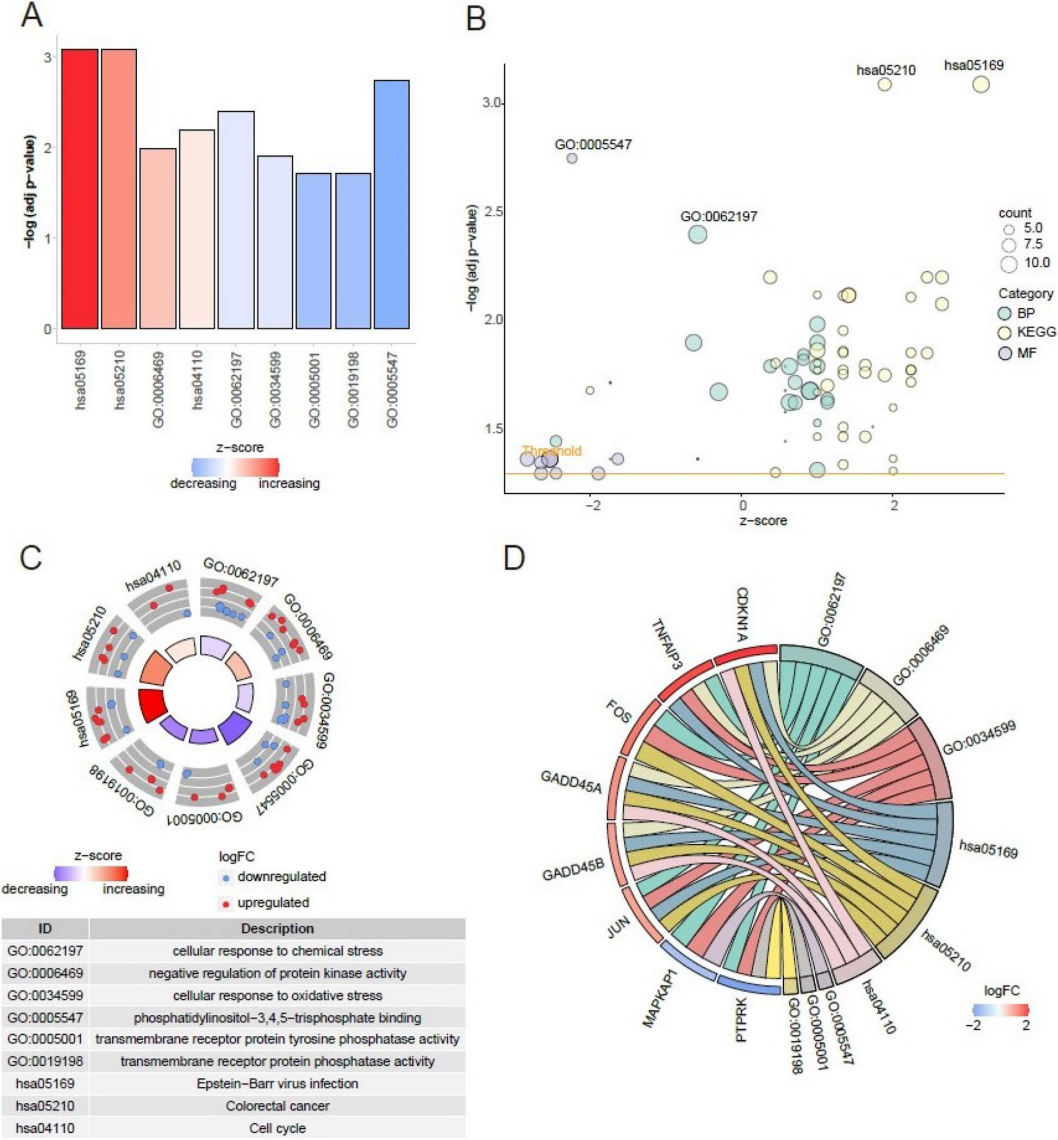
GO and KEGG analysis of DEGs between the experimental and control groups treated with cisplatin. **A**-**D** The histogram, bubble map, circle graph, and string diagram depict the outcomes of GO and KEGG enrichment analysis for DEGs. The histogram depicts enriched pathways, with increasing redness indicating higher up-regulated gene enrichment. The bubble diagram uses green for biological process pathways, yellow for KEGG pathways, and purple for molecular functional pathways, with bubble size representing the number of differentially expressed genes. In the outer scatter plot, red dots signify upregulation, blue dots indicate downregulation, while the inner circle bar chart illustrates the significance of enrichment results. Lastly, the chord diagram's left semicircle denotes genes, and the right semicircle denotes enriched pathways

We performed GO and KEGG (Tables S3 and S4) enrichment analysis on a set of 132 DEGs associated with cisplatin resistance, followed by the generation of a histogram (Fig. 2A), bubble plot (Fig. 2B), circular diagram (Fig. 2C), and chord diagram (Fig. 2D). The primary enrichment pathway of differentially expressed genes associated with cisplatin resistance is GO:0062197, which participates in cellular response to chemical stimuli. Additionally, the following gene ontology terms were identified: GO:0006469 (negative regulation of protein kinase activity), GO:0034599 (cellular response to oxidative stress), GO:0005547 (phosphatidylinositol-3,4,5-triphosphate binding), GO:0005001 (transmembrane receptor protein tyrosine phosphatase activity), and GO:0019198 (transmembrane receptor protein phosphatase activity). Furthermore, hsa05169 is linked to Epstein-Barr virus infection, hsa05210 is associated with colorectal cancer, and hsa04110 is involved in cell cycle regulation.

### GSEA and GSVA enrichment analysis

**Fig. 3:**
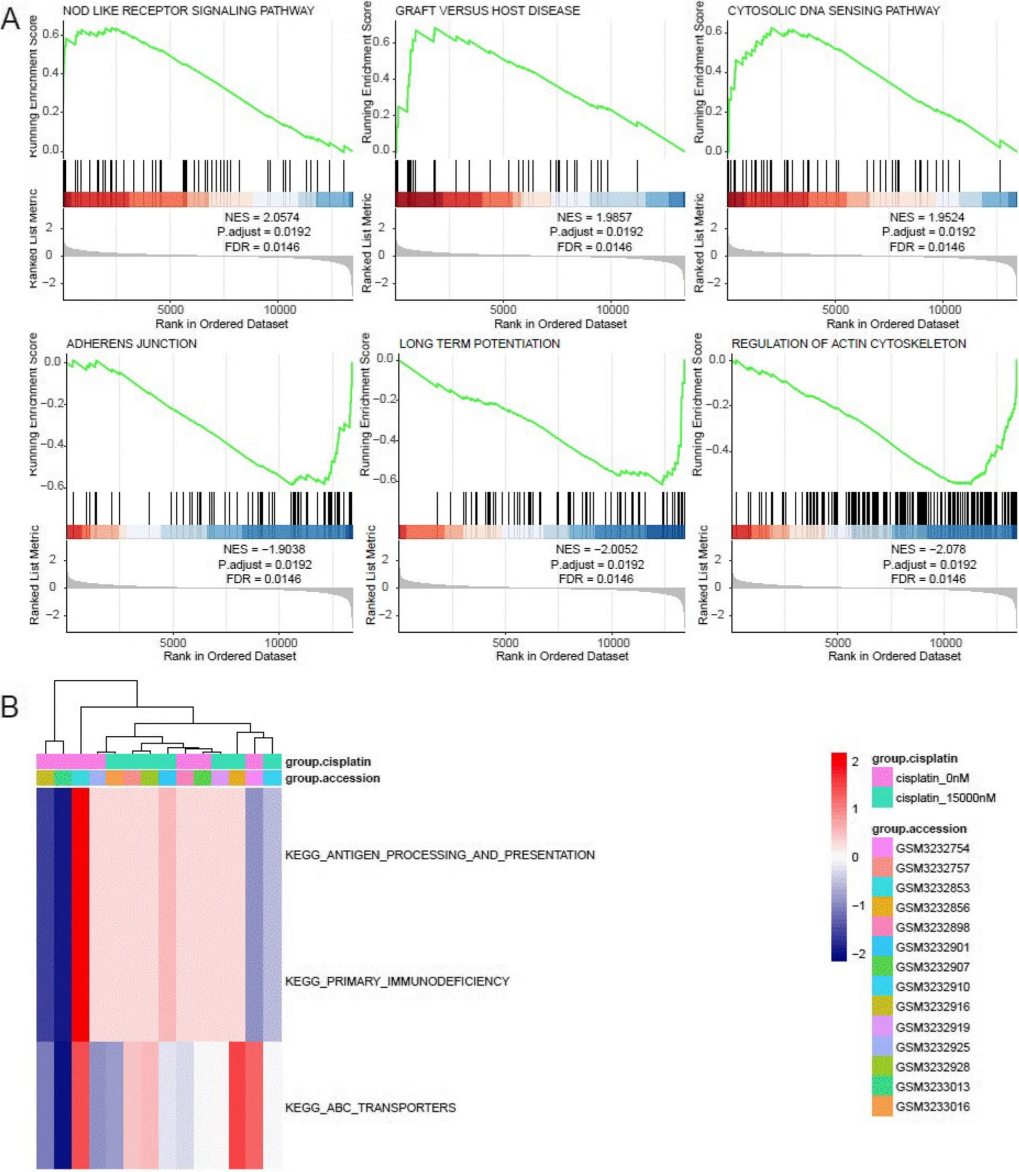
GSEA and GSVA enrichment analysis of cisplatin resistance related genes. **A** Pathway analysis using GSEA revealed significant enrichment of cisplatin resistance-related genes. **B** The distribution heat map of the significantly enriched pathways in each sample was analyzed using GSVA

Through GSEA, we obtained insights into the BP, CE, and MF associated with cisplatin resistance-related genes. These genes were found to be significantly enriched in key pathways such as Nod Like Receptor Signaling Pathway, Graft Versus Host Disease, Cytosolic Dna Sensing Pathway, Adherens Junction, Long Term Potentiation, And Regulation of Actin Cytoskeleton (Fig. 3A, Table S5). The distribution information of the enrichment fraction of cisplatin resistance-related genes obtained through GSVA analysis in each sample was visualized using heat maps. In comparison to the control group, the experimental group exhibited an irregular distribution pattern for the enrichment fraction of cisplatin resistance-related genes (Fig. 3B).

### A prognostic model of cisplatin resistance related genes was constructed based on TCGA data

**Fig. 4:**
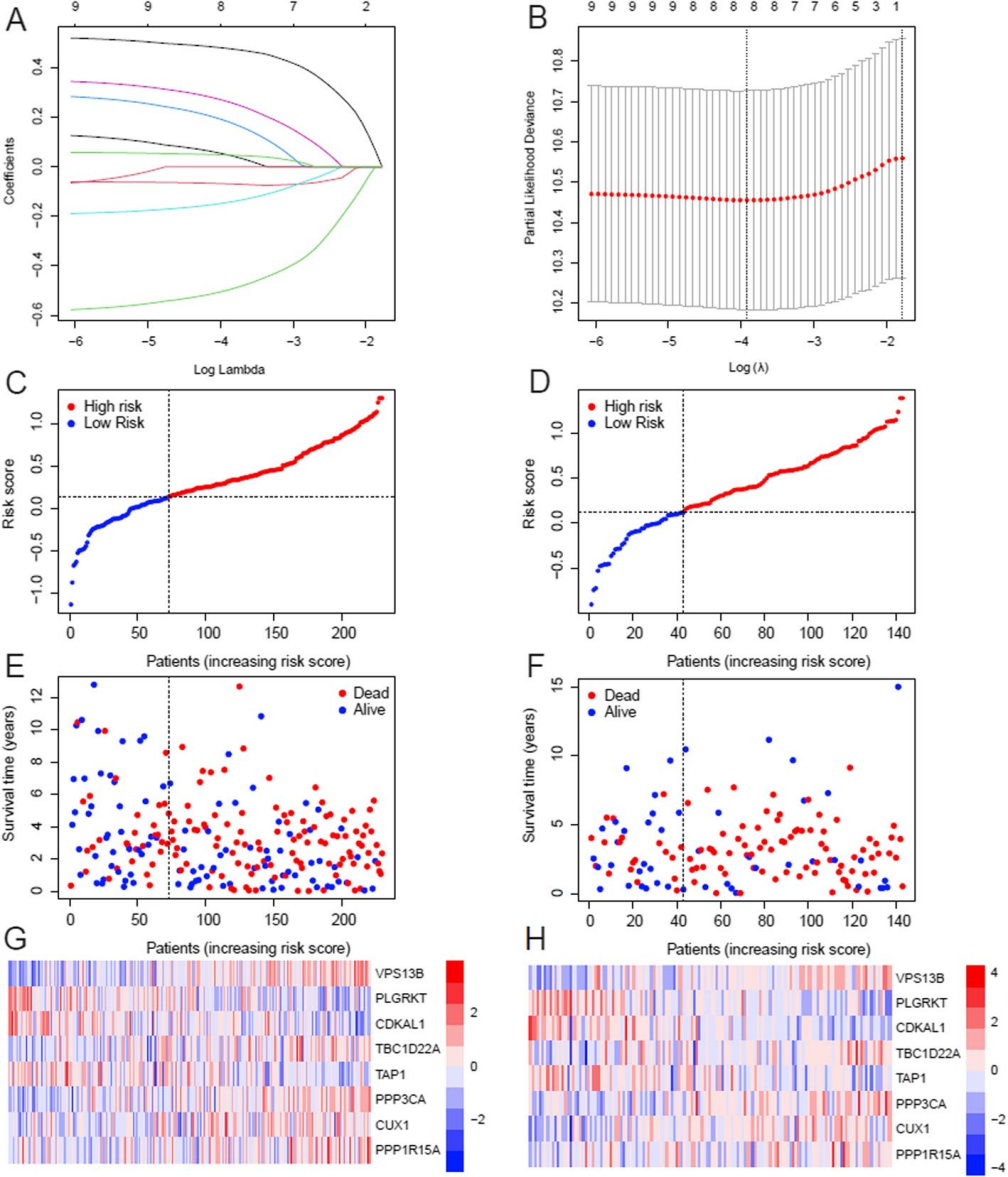
Prognosis model of cisplatin resistance related genes was constructed based on TCGA data. **A** The coefficient curve of LASSO regression analysis demonstrates the significant changes in lambda values for 9 genes associated with prognosis. **B** Ten-fold cross-validation plot. **C** The risk curve in the training dataset. **D** The risk curve in the test dataset. E. Scatter plot depicting survival state in the training dataset. **F** Scatter plot illustrating survival state in the test dataset. **G** Expression heat map displaying prognostic gene patterns in the training set. **H** Heat map showcasing prognostic gene expression patterns in the test set

In order to assess the prognostic correlation between genes associated with cisplatin resistance and ovarian cancer patients, we utilized TCGA-OV sequencing data and survival information to conduct a survival analysis of tumor samples for cisplatin resistance-related genes. We identified a total of 9 genes with significant prognostic value (*P* < 0.05). (Table S6). The LASSO-Cox regression algorithm was employed to establish the prognostic model (Fig. 4A, B), resulting in the identification of a risk model comprising eight genes (Table S7). Diseased samples were categorized into low and high-risk groups based on the median value of the risk score. The distribution of risk scores and survival status for both the training set (Table S8) and validation set (Table S9), along with an expression heat map illustrating the expression patterns of these eight genes, is shown in Fig. 4C-H.

### Survival analysis

**Fig.5:**
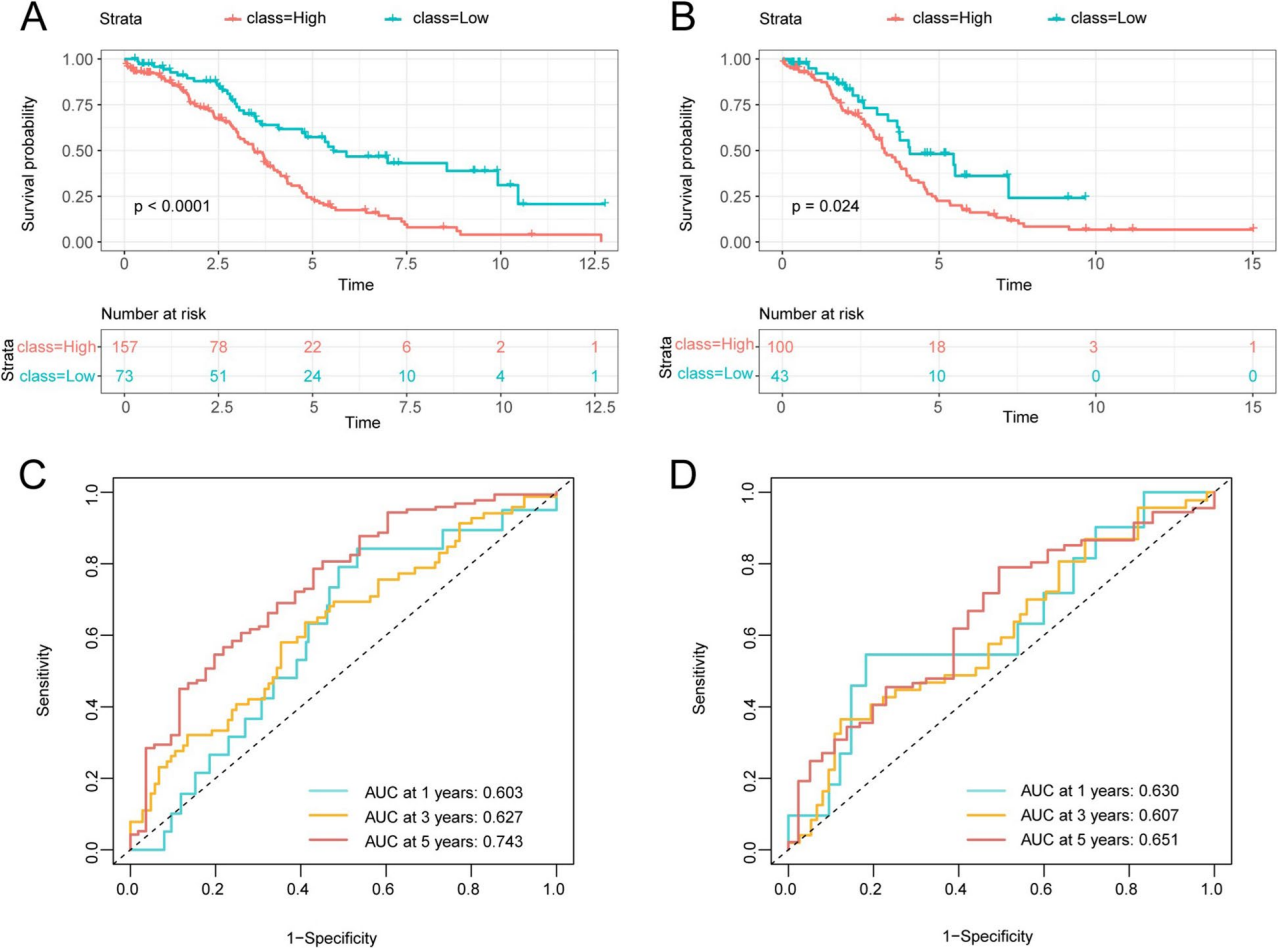
Survival analysis of ovarian cancer training set and test set. **A**, **B** The K-M survival analysis was conducted in both the training set (**A**) and validation set (**B**) to assess the high-low risk group. **C**, **D** ROC curves for risk scores were calculated from the training set (**C**) and test set (**D**) at 1-, 3-, and 5-year intervals

Survival analysis indicated significant differences in overall survival (OS) between the training group (Fig. 5A) and the validation group (Fig. 5B) within both high and low-risk groups (*P* < 0.05). The risk scores derived from the prognostic model showed an area under the curve (AUC) greater than 0.6 for 1-year, 3-year, and 5-year time points, suggesting a certain level of prognostic value for the model (Fig. 5C, D).

### Prognostic analysis based on clinical features

**Fig. 6:**
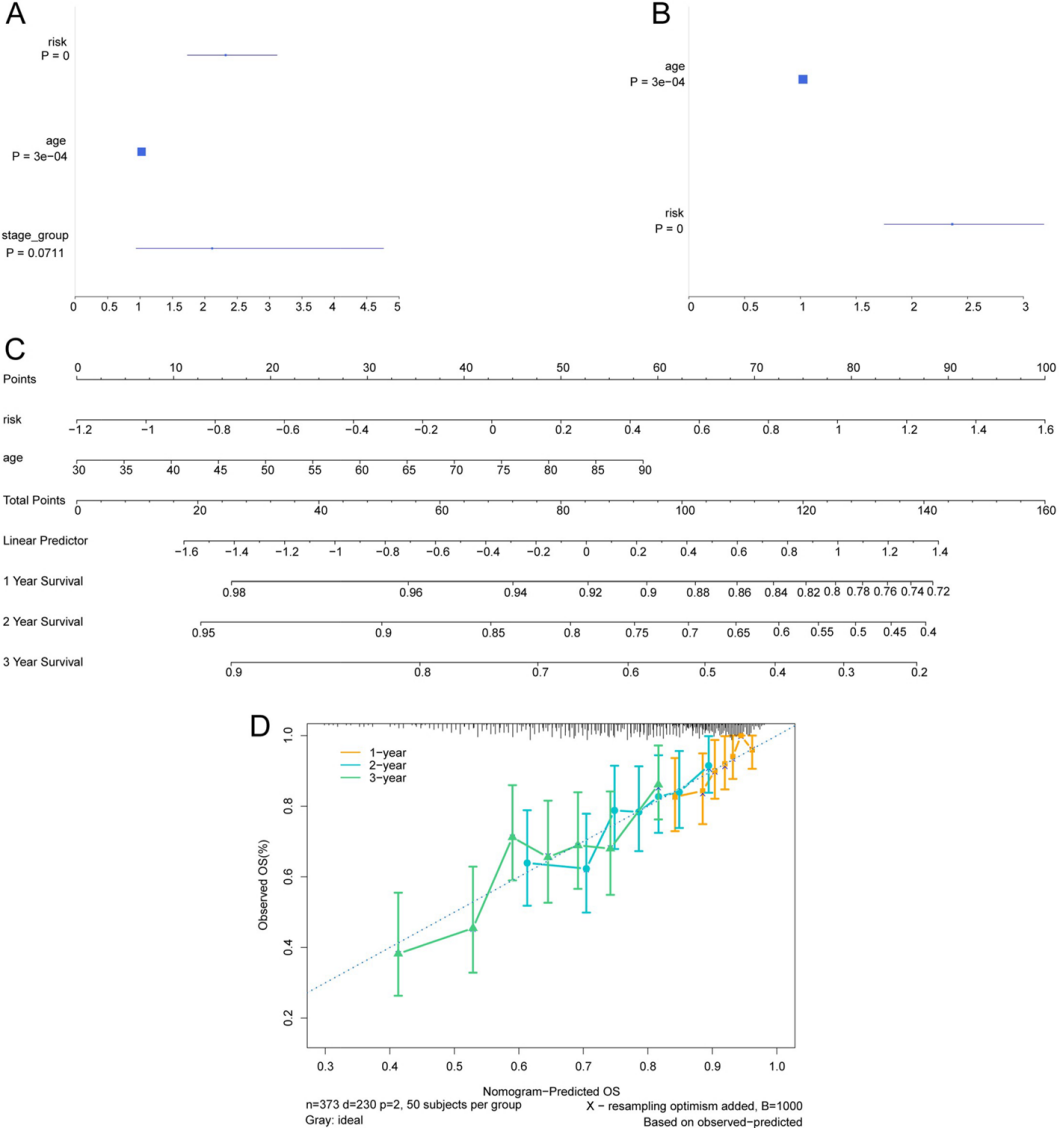
Prognosis analysis based on clinical features. **A** Unifactor COX regression forest map of clinical features. **B** Multivariate COX regression forest map incorporating clinical features. **C** Nomogram illustrating the clinical features. **D** Calibration curve for validation

Through univariate Cox regression analysis, we observed significant associations between survival and both risk scores and age (Fig. 6A). Moreover, multivariate Cox regression analysis identified age and risk scores as independent predictors of overall survival (OS) (Fig. 6B). Leveraging these factors, we constructed nomograms to evaluate the prognostic power of our model and generated a graphical representation (Fig. 6C), enabling quantification of individual's survival probabilities at 1, 2, and 3 years. The calibration curve demonstrated excellent concordance between predicted OS and actual observations across all time points (Fig. 6D).

### Immunoinfiltration analysis

**Fig. 7:**
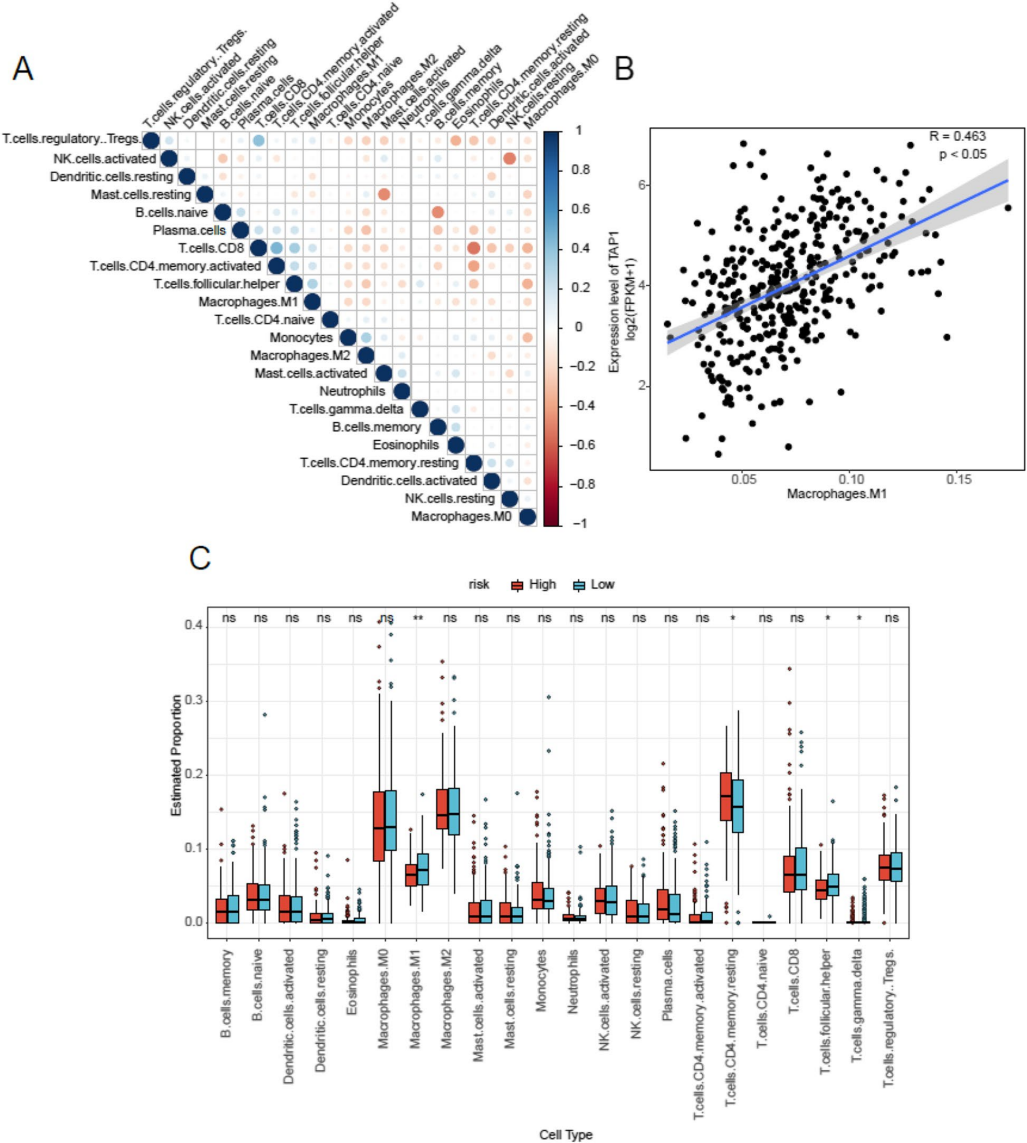
Analysis of immune infiltration in ovarian cancer samples. **A** Differences in the abundance of immune cells between two groups in ovarian cancer samples. **B** Correlation between TAP1 expression and the abundance of Macrophages M1 cells. **C** Association of immune cell infiltration with two groups

We quantified immune cell abundance in OC samples, obtaining values for 22 different types of immune cells. The correlation between various immune cell types in ovarian cancer samples was visually analyzed through a bubble heat map (Fig. 7A). This analysis helped us understand the level of correlation between different cell types and comprehend the characteristics of immune infiltration in ovarian cancer samples. For instance, the correlation between T cells CD8 and T cells regulatory Tregs, as well as T cells CD4 memory activated, is notably higher. There is low relevance observed between T cells CD8 and T cells CD4 memory resting. A scatter plot was employed to visually analyze the correlation between the prognostic gene and cell type, revealing a positive association between TAP1 gene expression and invasion degree of Macrophages M1 cell type (Fig. 7B). Furthermore, a boxplot was utilized to compare immune cell abundance in the high-risk and low-risk groups, demonstrating significant differences in Macrophages M1 cells, CD4 Memory Resting cells, T cells Follicular helper and T cells gamma delta (Fig. 7C).

### Drug sensitivity, tumor mutation analysis and prognostic gene chromosomal localization analysis

**Fig. 8:**
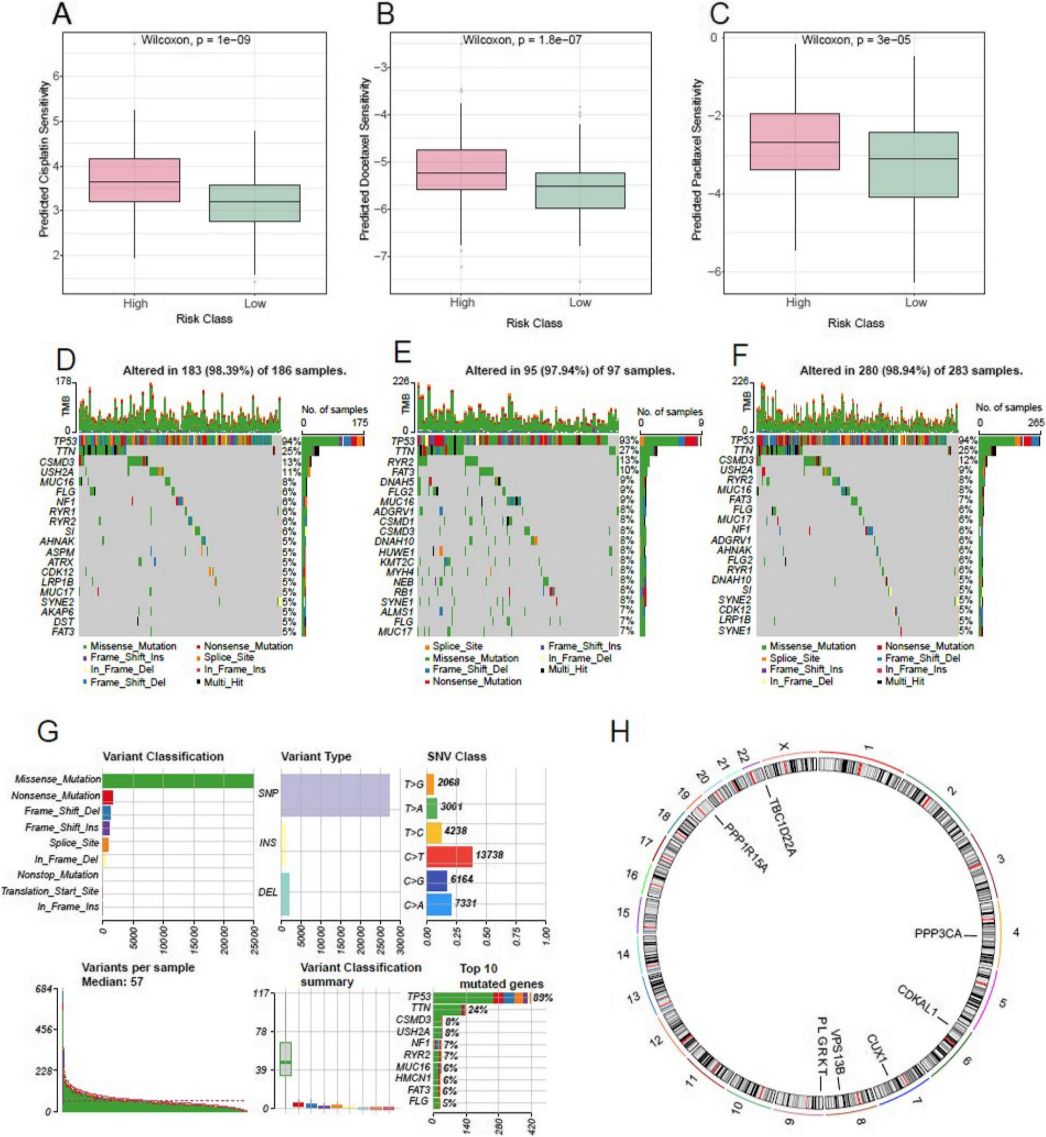
Drug sensitivity, TMB and Chromosome localization analysis of prognostic genes. **A**-**C** Differences in drug sensitivity between high and low-risk groups are observed. Boxplots demonstrate significant variations in drug sensitivity for Cisplatin (**A**), Docetaxel (**B**), and Paclitaxel (**C**) between the high and low-risk groups. **D** Mutation waterfall map illustrating differentially mutated genes in the high-risk group. **E** Mutation waterfall map displaying differentially mutated genes in the low-risk group. **F** Mutation waterfall map depicting differentially mutated genes in both high and low-risk groups. **G** Overview of genetic mutations found in ovarian cancer samples. **H** Chromosomal mapping of prognostic-related genes

Drug sensitivity analysis revealed prominent differences in the sensitivity of Cisplatin (Fig. 8A), Docetaxel (Fig. 8B), and Paclitaxel (Fig. 8C) between the high-risk and low-risk groups. We identified mutant genes in all TCGA-OV samples, with missense mutations being the predominant type of mutation observed (Fig. 8 D-G). Furthermore, we conducted chromosomal localization analysis to determine the coordinates of prognostic genes on chromosomes (Fig. 8H).

### ceRNA network analysis

**Fig. 9:**
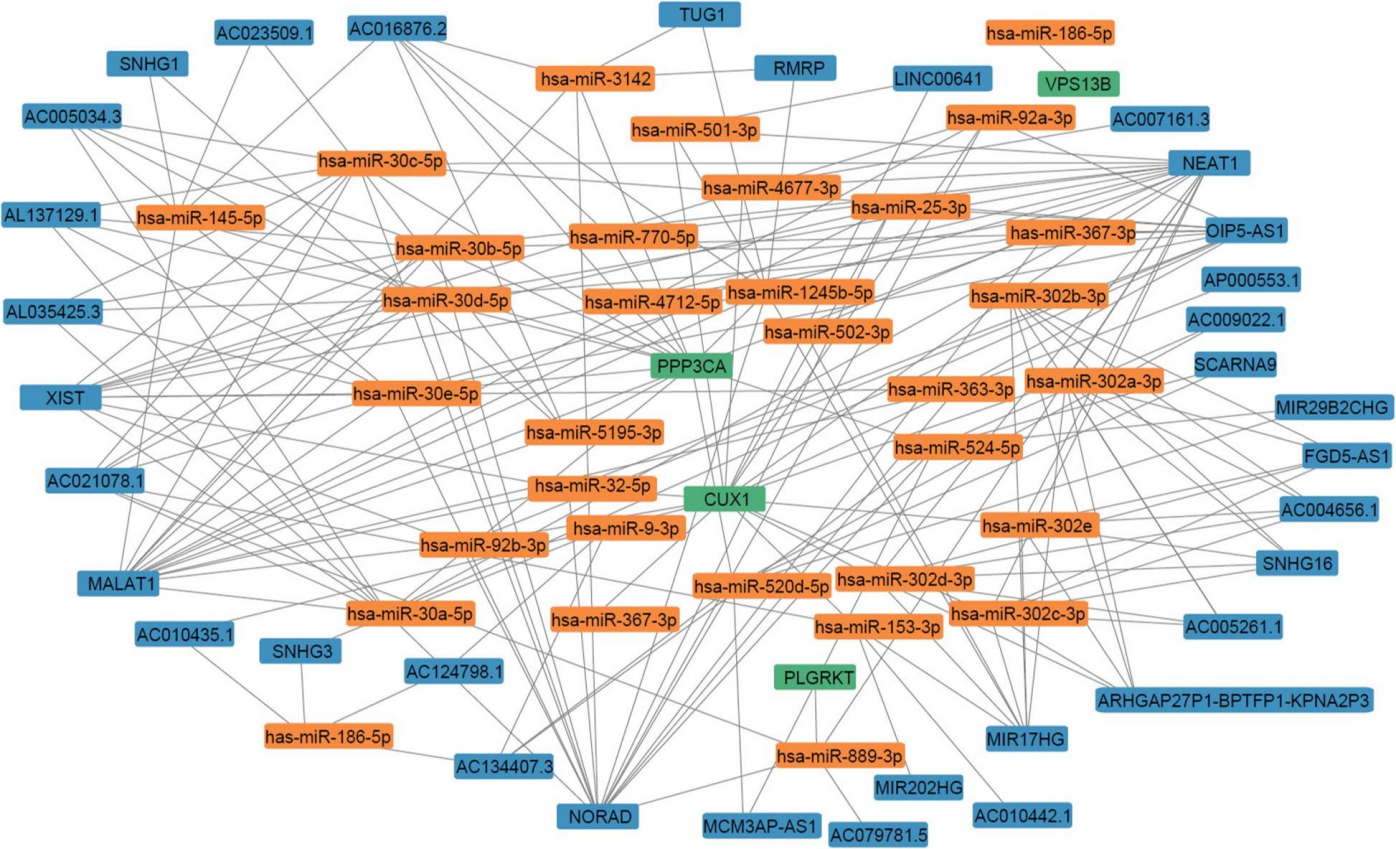
ceRNA network relationships among prognostic-related genes, microRNA, and lncRNA. The color green indicates the prognostic phase for genes, while orange represents microRNA and blue represents lncRNA

There are 8 genes associated with prognosis, namely VPS13B, PLGRKT, CDKAL1, TBC1D22A, TAP1, PPP3CA, CUX1 and PPP1R15A. All of these genes are mRNA. A total of 31 microRNAs targeting VPS13B, PPP3CA, CUX1 and PLGRKT genes were screened (Tables S10 S11, S12, 13). Then, we predicted the Long non-coding RNA (lncRNA) targeted by these 31 microRNA, and a total of 34 lncRNA were identified (Table S14). To visualize the network relationship between prognostic genes, microRNA, and lncRNA, we constructed a ceRNA network diagram (Fig. 9, Table S15).

### Validation of expression of prognostic related genes in ovarian cancer tissues

**Fig. 10:**
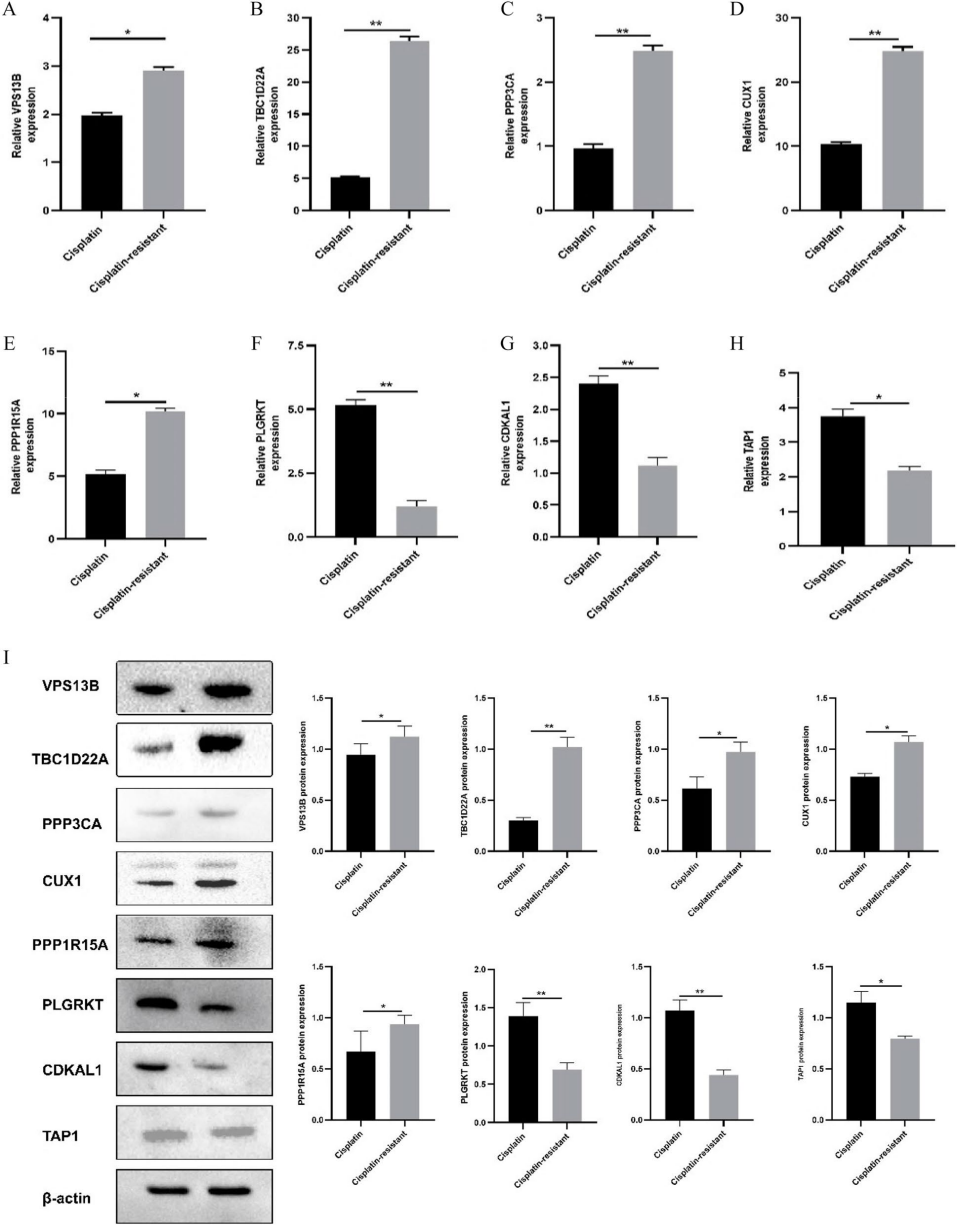
The expression of prognostic genes in ovarian and cisplatin-resistant ovarian cancers was assessed using QRT-PCR and Western blot analysis. **A** The expression of VPS13B was upregulated in the cisplatin-resistant group. **B** Increased expression of TBC1D22A was observed in the cisplatin-resistant group. **C** Enhanced expression of PPP3CA was detected in the cisplatin-resistant group. **D** CUX1 expression exhibited an increase in the cisplatin-resistant group. **E** Decreased expression of PPP1R15A was found in the cisplatin-resistant group. **F** The expression of PLGRKT was ownregulated in the cisplatin-resistant group. **G** CDKAL1 expression showed a decrease in the cisplatin-resistant group. **H** TAP1 expression demonstrated a reduction in the cisplatin-resistant group. **I** Western blot analysis revealed altered protein levels of VPS13B, PLGRKT, CDKAL1, TBC1D22A, TAP1, PPP3CA, CUX1 and PPP1R15A proteins between ovarian tissues and cisplatin-resistant tissues

The expressions of VPS13B, PLGRKT, CDKAL1, TBC1D22A, TAP1, PPP3CA, CUX1 and PPP1R15A in ovarian tissues of patients with OC and cisplatin-resistant OC were detected by QRT-PCR and Western Blot. The expression levels of VPS13B, TBC1D22A, PPP3CA, CUX1, and PPP1R15A genes were found to be up-regulated in ovarian tissue of cisplatin-resistant ovarian cancer patients compared to those with ovarian cancer. Conversely, the expression levels of PLGRKT, CDKAL1, and TAP1 genes were down-regulated in these patients' ovarian tissue (Fig. 10 A-I). These findings are consistent with the results obtained from bioinformatics analysis.

## Discussions

In recent years, there has been gradual advancement in the treatment of ovarian cancer, with surgery combined with chemotherapy emerging as the established standard approach. Early-stage patients can undergo comprehensive surgical intervention, while advanced-stage patients may benefit from tumor cell reduction procedures [10]. However, due to the predominance of advanced stage diagnoses for ovarian cancer cases, most patients require chemotherapy as an essential component of their treatment plan. Platinum-based combination chemotherapy stands as the primary therapeutic option for advanced ovarian cancer [10, 14]. However, the majority of patients undergoing chemotherapy eventually develop drug resistance, resulting in tumor recurrence and metastasis [14]. Approximately 20% of ovarian cancer patients exhibit inherent resistance to standard first-line platinum drug combination therapy, while platinum-resistant relapse cases account for approximately 25% of all relapse cases observed in clinical practice. Moreover, the prognosis for these cases is exceedingly poor [15], highlighting an urgent need for novel intervention targets.

The development of cisplatin resistance in OC chemotherapy encompasses a plethora of molecular alterations, including modifications in drug metabolism, mutations affecting drug targets, perturbations in DNA synthesis and repair mechanisms, initiation of cancer stem cell formation, immunosuppressive effects, deactivation of apoptotic genes, and activation of anti-apoptotic genes [14]. Based on multiple datasets obtained from GEO and TCGA databases, this study identified 132 differentially expressed genes related to cisplatin treatment in ovarian cancer cells, with 35 up-regulated and 97 down-regulated genes. The correlation analysis with cisplatin resistance genes identified eight hub genes, namely VPS13B, PLGRKT, CDKAL1, TBC1D22A, TAP1, PPP3CA, CUX1 and PPP1R15A.

Vacuolar Protein Sorting 13 Homolog B (VPS13B) is implicated in intracellular transport and subcellular localization, and mutations in this gene result in functional aberrations of the VPS13B protein, potentially impacting the normal functioning of diverse cell types and tissues [15]. In a study by Reika Iwakawa et al., frequent mutations and expression of genes were observed in small cell lung cancer including VPS13B [16]. In investigations concerning primary invasive breast cancer, aberrant methylation and transcription patterns of VPS13B have been implicated in the promotion of tumor suppressor gene inactivation or oncogene activation [17]. The PLGRKT receptor exhibits a distinctive structure and its proteolytic activity plays a crucial role in various physiological and pathological processes, encompassing inflammation, tumorigenesis, metastasis, fibrinolysis, cytokine induction, and activity release [18]. Plasminogen primarily regulates the inflammatory response by facilitating the recruitment, migration, and aggregation of plasminogen-dependent monocytes and macrophages [19]. In recent years, numerous studies have also demonstrated the pivotal role of plasminogen receptors in the regulation of tumor microenvironment. Lindsey A. Miles et al., for the first time, investigated the expression of PLGRKT in human breast cancer, wherein invasive ductal carcinoma exhibited the highest expression level. The phenomenon leads to degradation of fibrin and extracellular matrix, thereby promoting tumor progression [20]. CDKAL1 acts as a tRNA-modified methylthiotransferase, facilitating the production of cytokines that are characteristic of cancer stem cells. Huang et al. have demonstrated the essential role of CDKAL1 in maintaining stem cell-like cytokine profiles across various common cancers such as rhabdomyosarcoma, melanoma, liver cancer, stomach cancer and glioma [21, 22]. Moreover, they observed a correlation between elevated expression levels of CDKAL1 and unfavorable prognosis. TBC1D22A is a protein localized in the Golgi apparatus that plays a crucial role in preserving the integrity of the Golgi membrane and has been implicated in the pathogenesis of liver cancer, epilepsy and other diseases [23]. Transporter associated with antigen processing 1 (TAP1) is a crucial molecule responsible for the processing and presentation of tumor-associated antigens. Aberrant expression of TAP1 has been observed in various tumor types and is known to impact multidrug resistance in human cancer cell lines during chemotherapy [24, 25]. Qianxia Tan et al. discovered that high levels of TAP1 expression serve as an independent prognostic indicator for ovarian cancer patients, correlating with favorable outcomes [24, 26]. The alpha isozyme of protein phosphatase 3, catalytic subunit (PPP3CA) represents as a calmodulin-regulated serine-threonine phosphatase. Variants in PPP3CA have been implicated in the development of early-onset, refractory epilepsy [23, 25, 27]. Aberrant expression of PPP3CA has also been observed in advanced multiple myeloma (MM), suggesting a potential association between elevated levels of PPP3CA and MM pathogenesis [28]. Furthermore, within an immune and iron-death-related risk score model for ovarian cancer patients developed by Chunyan Wei et al., PPP3CA has been identified as a prognostic factor aiding in predicting patient response to immunotherapy [29]. These findings align with our study results, emphasizing the significance of PPP3CA as a pivotal prognostic factor in ovarian cancer. CUX1 (CUT-like homeobox 1) is identified as a haploid tumor suppressor associated with both tumor inhibition and progression. [30]. Studies have confirmed that the circRNA derived from Cux1, encoding protein P113, drives neuroblastoma (NB) progression by facilitating the trans-activation of ZRF1/BRD4. It exhibits high expression in NB cells and promotes their proliferation, invasion, and metastasis [31]. Investigations on pancreatic neurosecretory tumors (pan-NET) have demonstrated that CUX1 serves as a prognostic marker post PanNET surgery and facilitates in vitro tumor progression through enhanced proliferation and angiogenesis [32].

Functional analysis using GO and KEGG indicated that these differentially expressed genes are primarily involved in the negative regulation of protein kinase activity, oxidative stress response, chemical cell response. Additionally, they are associated with Epstein-Barr virus infection, colorectal cancer, and cell cycle. Meanwhile, GSEA enrichment analysis revealed significant associations of these differential genes with long-term potentiation, regulation of actin cytoskeleton, graft-versus-host disease, and cellular DNA sensing pathways.

Furthermore, employing the LASSO-cox regression algorithm, we constructed a risk model comprising 8 differential genes. Survival analysis results demonstrated that the high-risk group exhibited a significantly lower survival rate compared to the low-risk group (*P* < 0.05). Analysis of clinical characteristics identified age and risk score as independent prognostic factors for predicting survival. Further investigation into immune cell abundance disparities between high-risk and low-risk groups was conducted through immunoinfiltration analysis. In the results from these two groups, significant differences were observed in the levels of macrophages, specifically M1, T cells CD4 memory resting, T cells follicular helper, and T cells gamma delta. The expression of TAP1 positively correlates with Macrophages M1 cell type. The findings reveal that in the group exhibiting heightened expression of CA125, a serum tumor marker associated with various cancers including ovarian, endometrial, and bladder cancers, there were elevated levels of M2 macrophage marker, CD163, as well as the regulatory T-cell (Treg) marker, FOXP3, compared to the group with lower CA125 expression. This indicates that individuals with increased CA125 expression in bladder cancer tend to possess a tumor microenvironment characterized by immunosuppression [33]. Our results are congruent with this observation, indicating variances in the immune milieu between high-risk and low-risk patients with cancer. Risk prediction models accurately forecast survival outcomes in patients with cisplatin-resistant OC. Through biological information screening, RT-qPCR and WB verification, it was found that VPS13B, TBC1D22A, PPP3CA, CUX1 and PPP1R15A were highly expressed in cisplatin-resistant tissues of ovarian cancer, while PLGRKT, CDKAL1 and TAP1 were low expressed. The conclusion is consistent with the previous conclusions and enriches their research [23, 29].

In this study, we conducted the first screening of differential genes between OC cells and cisplatin-resistant OC cells. However, there are certain limitations in our study. Firstly, the dataset included only 14 patients, which may not provide sufficient evidence to accurately assess the predictive accuracy of the prognostic model. Additionally, our study relies on previous research data; thus, further experimental validation is necessary to clarify the pathogenesis of these genes in the disease and improve their predictive power for clinical applications.

## Conclusion

Through bioinformatics analysis of EMS expression profile data, we identified 132 DEGs and 8 prognostic genes. Subsequently, by conducting ceRNA network analysis, VPS13B, TBC1D22A, PPP3CA, CUX1, and PPP1R15A were identified as poor prognostic genes associated with cisplatin resistance in OC. Conversely, PLGRKT, CDKAL1, and TAP1 were found to be good prognostic genes. These findings hold significant implications for the development of novel molecular therapeutic targets and provide a solid theoretical foundation for further investigation into their underlying molecular mechanisms.

### Supplementary Information

**Supplementary Material 1.**

**Supplementary Material 2.**

**Supplementary Material 3.**

**Supplementary Material 4.**

**Supplementary Material 5.**

**Supplementary Material 6.**

**Supplementary Material 7.**

**Supplementary Material 8.**

**Supplementary Material 9.**

**Supplementary Material 10.**

**Supplementary Material 11.**

**Supplementary Material 12.**

**Supplementary Material 13.**

**Supplementary Material 14.**

**Supplementary Material 15.**

**Supplementary Material 16.**

## Data Availability

The datasets generated and analysed during the current study are available in the GEO repository, (https://www.ncbi.nlm.nih.gov/).

## References

[1] Wang Z, Guo E, Yang B, Xiao R, Lu F, You L, Chen G. Trends and age-period-cohort effects on mortality of the three major gynecologic cancers in China from 1990 to 2019: cervical, ovarian and uterine cancer. Gynecol Oncol. 2021;163(2):358–63. 34507827 10.1016/j.ygyno.2021.08.029

[2] Yi M, Li T, Niu M, Luo S, Chu Q, Wu K. Epidemiological trends of women’s cancers from 1990 to 2019 at the global, regional, and national levels: a population-based study. Biomark Res. 2021;9(1):55. 34233747 10.1186/s40364-021-00310-y PMC8261911

[3] Zhang R, Siu MKY, Ngan HYS, Chan KKL. Molecular biomarkers for the early detection of ovarian cancer. Int J Mol Sci. 2022;23(19):12041. 36233339 10.3390/ijms231912041 PMC9569881

[4] Ogundipe OD, Olajubutu O, Adesina SK. Targeted drug conjugate systems for ovarian cancer chemotherapy. Biomed Pharmacother. 2023;165:115151. 37473683 10.1016/j.biopha.2023.115151

[5] Kuroki L, Guntupalli SR. Treatment of epithelial ovarian cancer. BMJ. 2020;371:m3773. 33168565 10.1136/bmj.m3773

[6] Lheureux S, Gourley C, Vergote I, Oza AM. Epithelial ovarian cancer. Lancet. 2019;393(10177):1240–53. 30910306 10.1016/S0140-6736(18)32552-2

[7] Baert T, Ferrero A, Sehouli J, O’Donnell DM, González-Martín A, et al. The systemic treatment of recurrent ovarian cancer revisited. Ann Oncol. 2021;32(6):710–25. 33675937 10.1016/j.annonc.2021.02.015

[8] Sundar S, Neal RD, Kehoe S. Diagnosis of ovarian cancer. BMJ. 2015;351:h4443. 26328593 10.1136/bmj.h4443

[9] Menon U, Karpinskyj C, Gentry-Maharaj A. Ovarian cancer prevention and screening. Obstet Gynecol. 2018;131(5):909–27. 29630008 10.1097/AOG.0000000000002580

[10] Chun J. Isoalantolactone suppresses glycolysis and Resensitizes cisplatin-based chemotherapy in cisplatin-resistant ovarian cancer cells. Int J Mol Sci. 2023;24(15):12397. 37569773 10.3390/ijms241512397 PMC10419319

[11] Li F, Zheng Z, Chen W, Li D, Zhang H, Zhu Y, Mo Q, Zhao X, Fan Q, Deng F, Han C, Tan W. Regulation of cisplatin resistance in bladder cancer by epigenetic mechanisms. Drug Resist Updat. 2023;68:100938. 36774746 10.1016/j.drup.2023.100938

[12] Wang Z, Chen W, Zuo L, Xu M, Wu Y, Huang J, Zhang X, Li Y, Wang J, Chen J, Wang H, Sun H. The Fibrillin-1/VEGFR2/STAT2 signaling axis promotes chemoresistance via modulating glycolysis and angiogenesis in ovarian cancer organoids and cells. Cancer Commun (Lond). 2022;42(3):245–65. 35234370 10.1002/cac2.12274 PMC8923131

[13] Nie S, Zhang L, Liu J, Wan Y, Jiang Y, Yang J, Sun R, Ma X, Sun G, Meng H, Xu M, Cheng W. ALKBH5-HOXA10 loop-mediated JAK2 m6A demethylation and cisplatin resistance in epithelial ovarian cancer. J Exp Clin Cancer Res. 2021;40(1):284.10. 34496932 10.1186/s13046-021-02088-1 PMC8425158

[14] Iwakawa R, Kohno T, Totoki Y, Shibata T, Tsuchihara K, Mimaki S, et al. Expression and clinical significance of genes frequently mutated in small cell lung cancers defined by whole exome/RNA sequencing. Carcinogenesis. 2015;36(6):616–21. 25863124 10.1093/carcin/bgv026 PMC4462675

[15] Hao L, Wang JM, Liu BQ, Yan J, Li C, Jiang JY, et al. m6A-YTHDF1-mediated TRIM29 upregulation facilitates the stem cell-like phenotype of cisplatin-resistant ovarian cancer cells. Biochim Biophys Acta Mol Cell Res. 2021;1868(1):118878. 33011193 10.1016/j.bbamcr.2020.118878

[16] Parris TZ, Kovács A, Hajizadeh S, Nemes S, Semaan M, Levin M, et al. Frequent MYC coamplification and DNA hypomethylation of multiple genes on 8q in 8p11-p12-amplified breast carcinomas. Oncogenesis. 2014;3(3):e95. 24662924 10.1038/oncsis.2014.8 PMC4038389

[17] Miles LA, Vago JP, Sousa LP, Parmer RJ. Functions of the plasminogen receptor Plg-RKT. J Thromb Haemost. 2020;18(10):2468–81. 32662180 10.1111/jth.15014 PMC7722214

[18] Godier A, Hunt BJ. Plasminogen receptors and their role in the pathogenesis of inflammatory, autoimmune and malignant disease. J Thromb Haemost. 2013;11(1):26–34. 23140188 10.1111/jth.12064

[19] Miles LA, Krajewski S, Baik N, Parmer RJ, Mueller BM. Plg-RKT Expression in Human Breast Cancer Tissues. Biomolecules. 2022;12(4):503. 35454092 10.3390/biom12040503 PMC9028288

[20] Huang R, Yamamoto T, Nakata E, Ozaki T, Kurozumi K, Wei F, Tomizawa K, Fujimura A. CDKAL1 Drives the Maintenance of Cancer Stem-Like Cells by Assembling the eIF4F Translation Initiation Complex. Adv Sci (Weinh). 2023;10(12):e2206542. 36786012 10.1002/advs.202206542 PMC10131790

[21] Nalesnik MA, Tseng G, Ding Y, Xiang GS, Zheng ZL, Yu Y, et al. Gene deletions and amplifications in human hepatocellular carcinomas: correlation with hepatocyte growth regulation. Am J Pathol. 2012;180(4):1495–508. 22326833 10.1016/j.ajpath.2011.12.021 PMC3657620

[22] Palmer CJ, Bruckner RJ, Paulo JA, Kazak L, Long JZ, Mina AI, et al. Cdkal1, a type 2 diabetes susceptibility gene, regulates mitochondrial function in adipose tissue. Mol Metab. 2017;6(10):1212–25. 29031721 10.1016/j.molmet.2017.07.013 PMC5641635

[23] Tabassum A, Samdani MN, Dhali TC, Alam R, Ahammad F, Samad A, Karpiński TM. Transporter associated with antigen processing 1 (TAP1) expression and prognostic analysis in breast, lung, liver, and ovarian cancer. J Mol Med (Berl). 2021;99(9):1293–309. 34047812 10.1007/s00109-021-02088-w PMC8367907

[24] Tan Q, Liu H, Xu J, Mo Y, Dai F. Integrated analysis of tumor-associated macrophage infiltration and prognosis in ovarian cancer. Aging (Albany NY). 2021;13(19):23210–32. 34633990 10.18632/aging.203613 PMC8544311

[25] Li X, Zeng S, Ding Y, Nie Y, Yang M. Comprehensive Analysis of the Potential Immune-Related Biomarker Transporter Associated With Antigen Processing 1 That Inhibits Metastasis and Invasion of Ovarian Cancer Cells. Front Mol Biosci. 2021;8:763958. 34957213 10.3389/fmolb.2021.763958 PMC8702961

[26] Panneerselvam S, Wang J, Zhu W, Dai H, Pappas JG, Rabin R, et al. PPP3CA truncating variants clustered in the regulatory domain cause early-onset refractory epilepsy. Clin Genet. 2021;100(2):227–33. 33963760 10.1111/cge.13979 PMC11698261

[27] Campbell JD, Alexandrov A, Kim J, Wala J, Berger AH, Pedamallu CS, Shukla SA, Guo G, Brooks AN, Murray BA, Imielinski M, Hu X, Ling S, Akbani R, Rosenberg M, et al. Distinct patterns of somatic genome alterations in lung adenocarcinomas and squamous cell carcinomas. Nat Genet. 2016;48(6):607–16. 27158780 10.1038/ng.3564 PMC4884143

[28] Imai Y, Maru Y, Tanaka J. Action mechanisms of histone deacetylase inhibitors in the treatment of hematological malignancies. Cancer Sci. 2016;107(11):1543–9. 27554046 10.1111/cas.13062 PMC5132279

[29] Wei C, Zhao G, Gao M, Liu Y, Lei P, Cao T. Construction of an immunity and Ferroptosis-related risk score model to predict ovarian cancer clinical outcomes and immune microenvironment. Front Biosci (Landmark Ed). 2023;28(1):4. 36722270 10.31083/j.fbl2801004

[30] Ramdzan ZM, Nepveu A. CUX1, a haploinsufficient tumour suppressor gene overexpressed in advanced cancers. Nat Rev Cancer. 2014;14(10):673–82. 25190083 10.1038/nrc3805

[31] Yang F, Hu A, Guo Y, Wang J, Li D, Wang X, Jin S, Yuan B, Cai S, Zhou Y, Li Q, Chen G, Gao H, Zheng L, Tong Q. p113 isoform encoded by CUX1 circular RNA drives tumor progression via facilitating ZRF1/BRD4 transactivation. Mol Cancer. 2021;20(1):123. 34579723 10.1186/s12943-021-01421-8 PMC8474885

[32] Krug S, Weissbach J, Blank A, Perren A, Haybaeck J, Fendrich V, Rinke A, et al. CUX1-Transcriptional Master Regulator of Tumor Progression in Pancreatic Neuroendocrine Tumors. Cancers (Basel). 2020;12(7):1957. 32707646 10.3390/cancers12071957 PMC7409270

[33] Yamashita T, Higashi M, Sugiyama H, Morozumi M, Momose S, Tamaru JI. Cancer antigen 125 expression enhances the gemcitabine/cisplatin-resistant tumor microenvironment in bladder cancer. Am J Pathol. 2023;193(3):350–61. 36586479 10.1016/j.ajpath.2022.12.005

