# Supplementary figures and images for: Identification and validation of genes associated with prognosis of cisplatin-resistant ovarian cancer
Source: BMC Cancer. 2024 Aug 5;24:508. doi: 10.1186/s12885-024-12264-z (PMC11302001; doi:10.1186/s12885-024-12264-z)

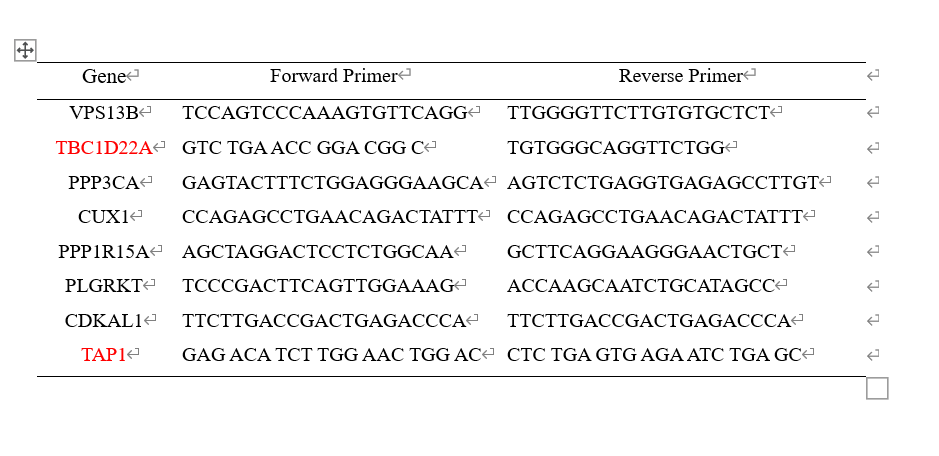

Supplement: Supplementary file 1 — Supplementary Material 1. [file 12885_2024_12264_MOESM1_ESM.png]
