# Supplementary material for: Identification and validation of genes associated with prognosis of cisplatin-resistant ovarian cancer
Source: BMC Cancer. 2024 Aug 5;24:508. doi: 10.1186/s12885-024-12264-z (PMC11302001; doi:10.1186/s12885-024-12264-z)
Supplement: Supplementary file 16 — Supplementary Material 16. [file 12885_2024_12264_MOESM16_ESM.pptx]

## Slide 1
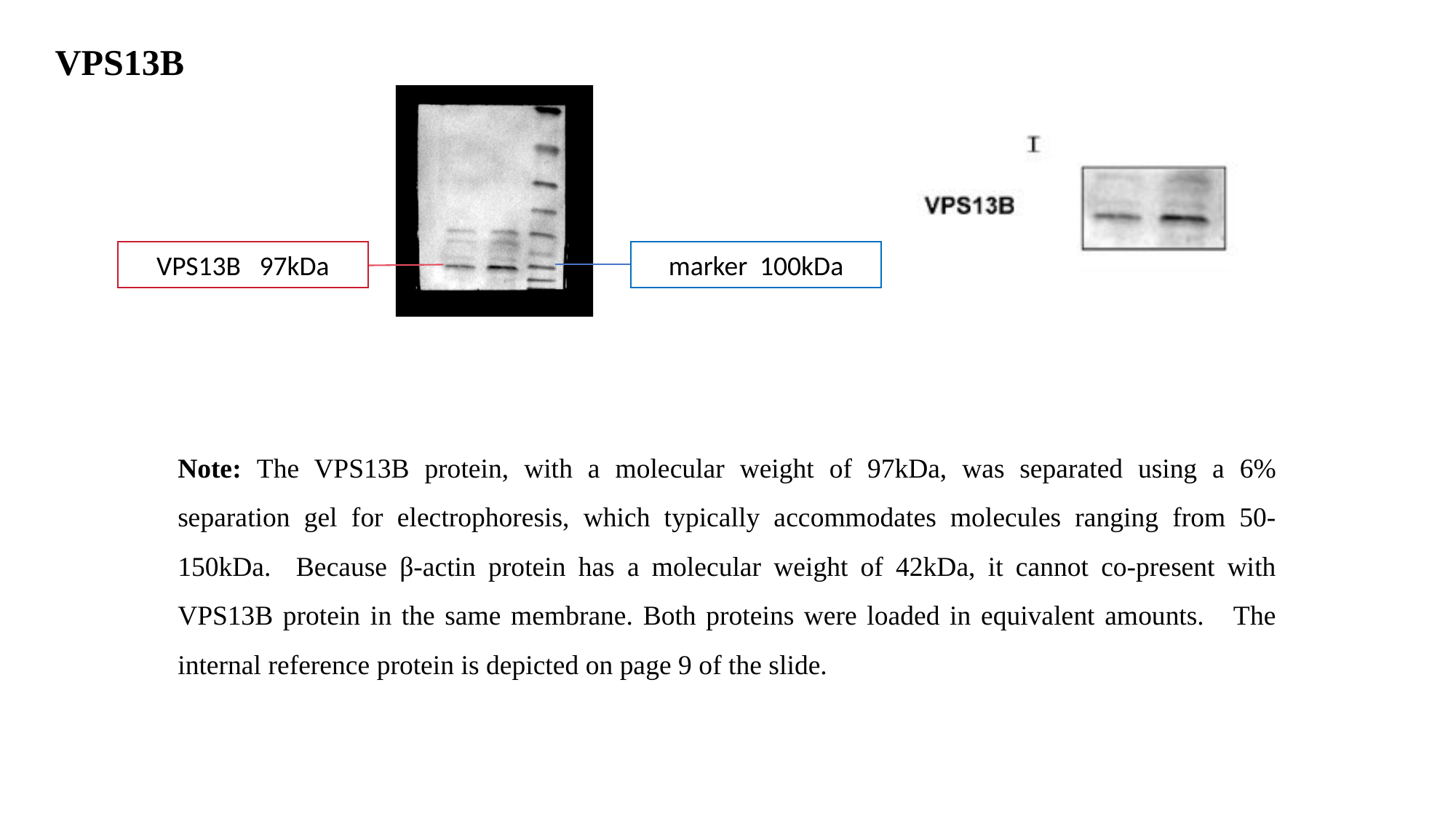

VPS13B
VPS13B 97kDa
marker 100kDa
Note: The VPS13B protein, with a molecular weight of 97kDa, was separated using a 6% separation gel for electrophoresis, which typically accommodates molecules ranging from 50-150kDa. Because β-actin protein has a molecular weight of 42kDa, it cannot co-present with VPS13B protein in the same membrane. Both proteins were loaded in equivalent amounts. The internal reference protein is depicted on page 9 of the slide.

## Slide 2
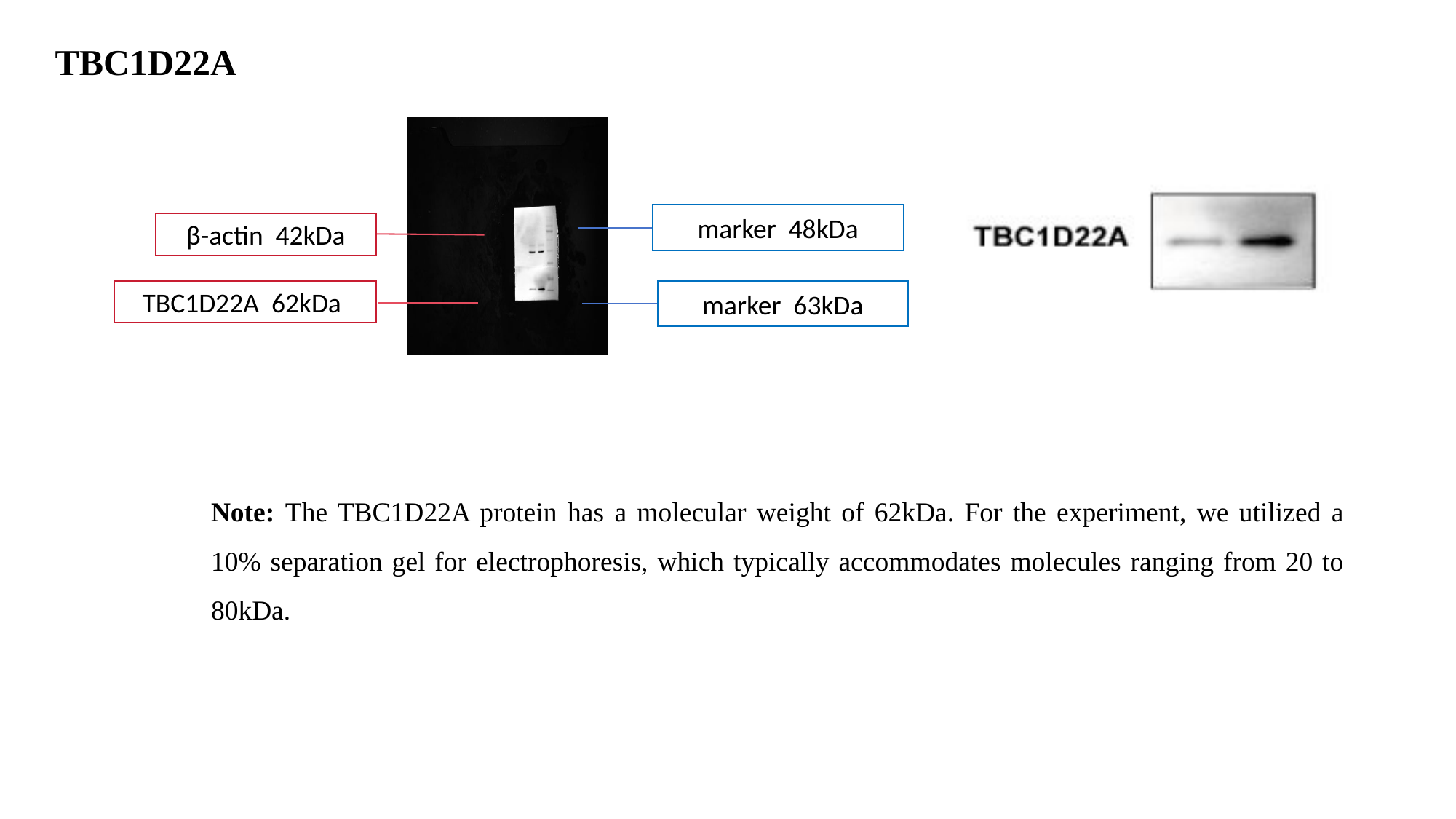

TBC1D22A
marker 48kDa
β-actin 42kDa
TBC1D22A 62kDa
marker 63kDa
Note: The TBC1D22A protein has a molecular weight of 62kDa. For the experiment, we utilized a 10% separation gel for electrophoresis, which typically accommodates molecules ranging from 20 to 80kDa.

## Slide 3
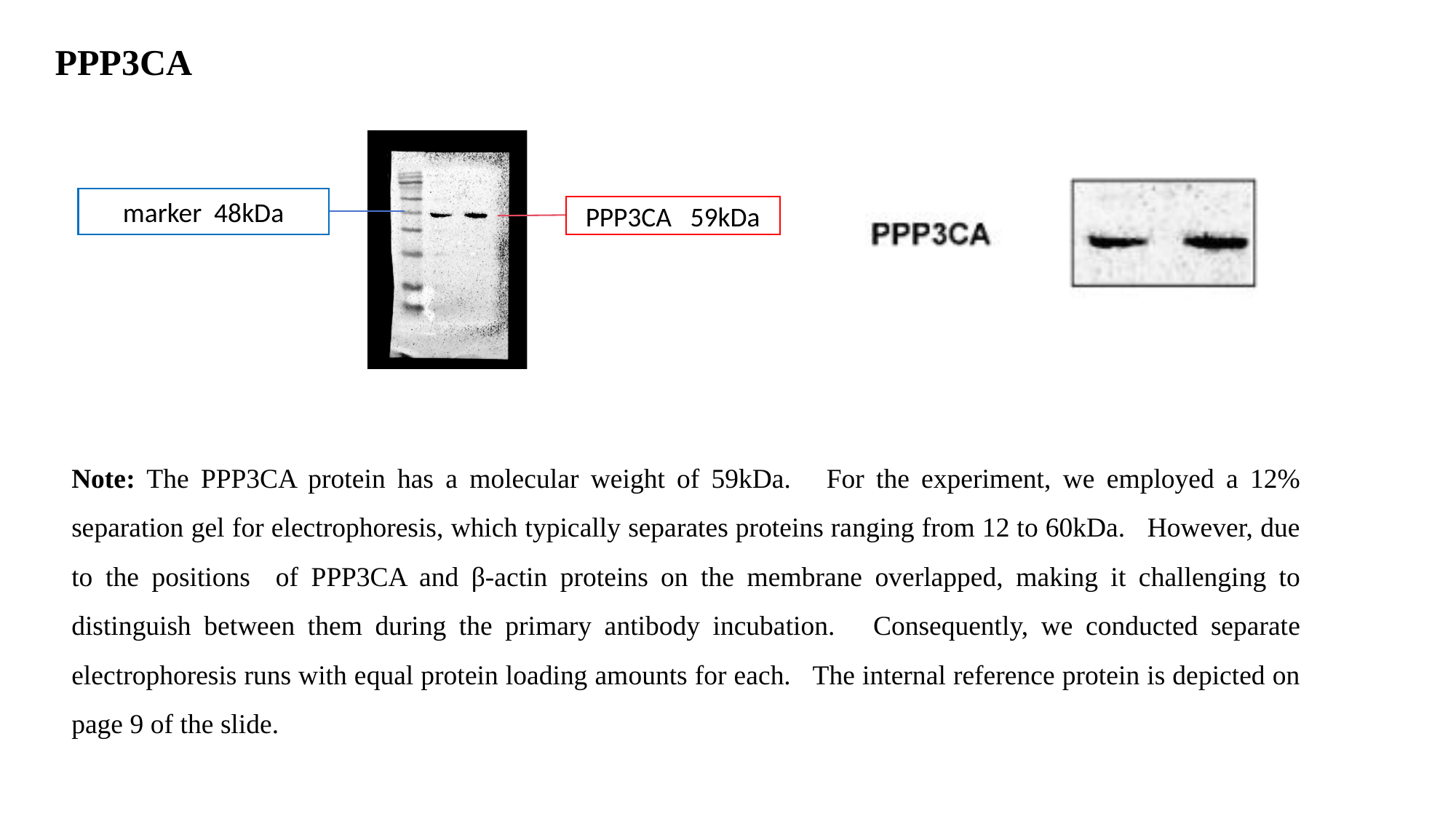

PPP3CA
marker 48kDa
PPP3CA 59kDa
Note: The PPP3CA protein has a molecular weight of 59kDa. For the experiment, we employed a 12% separation gel for electrophoresis, which typically separates proteins ranging from 12 to 60kDa. However, due to the positions of PPP3CA and β-actin proteins on the membrane overlapped, making it challenging to distinguish between them during the primary antibody incubation. Consequently, we conducted separate electrophoresis runs with equal protein loading amounts for each. The internal reference protein is depicted on page 9 of the slide.

## Slide 4
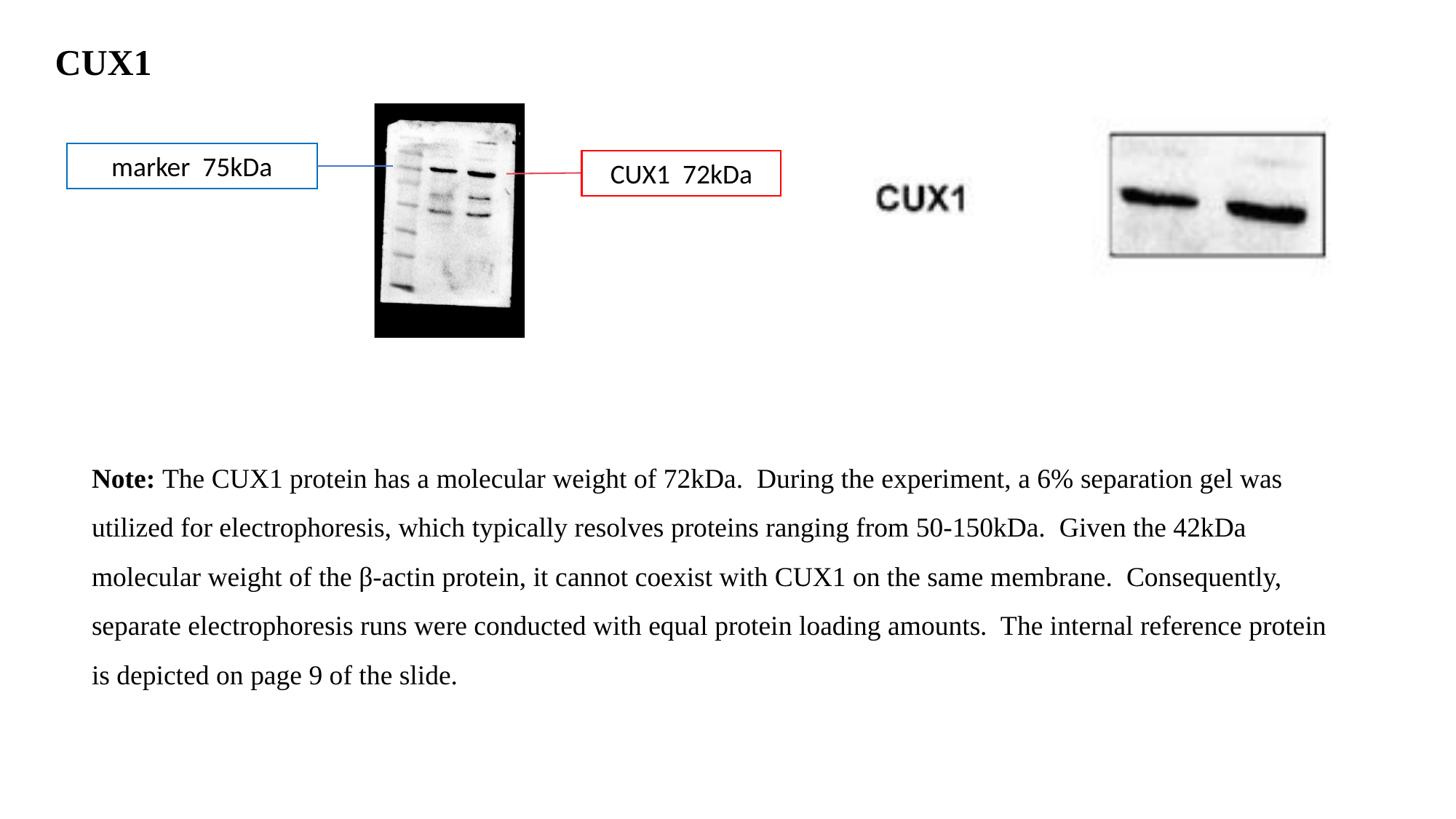

CUX1
marker 75kDa
CUX1 72kDa
Note: The CUX1 protein has a molecular weight of 72kDa. During the experiment, a 6% separation gel was utilized for electrophoresis, which typically resolves proteins ranging from 50-150kDa. Given the 42kDa molecular weight of the β-actin protein, it cannot coexist with CUX1 on the same membrane. Consequently, separate electrophoresis runs were conducted with equal protein loading amounts. The internal reference protein is depicted on page 9 of the slide.

## Slide 5
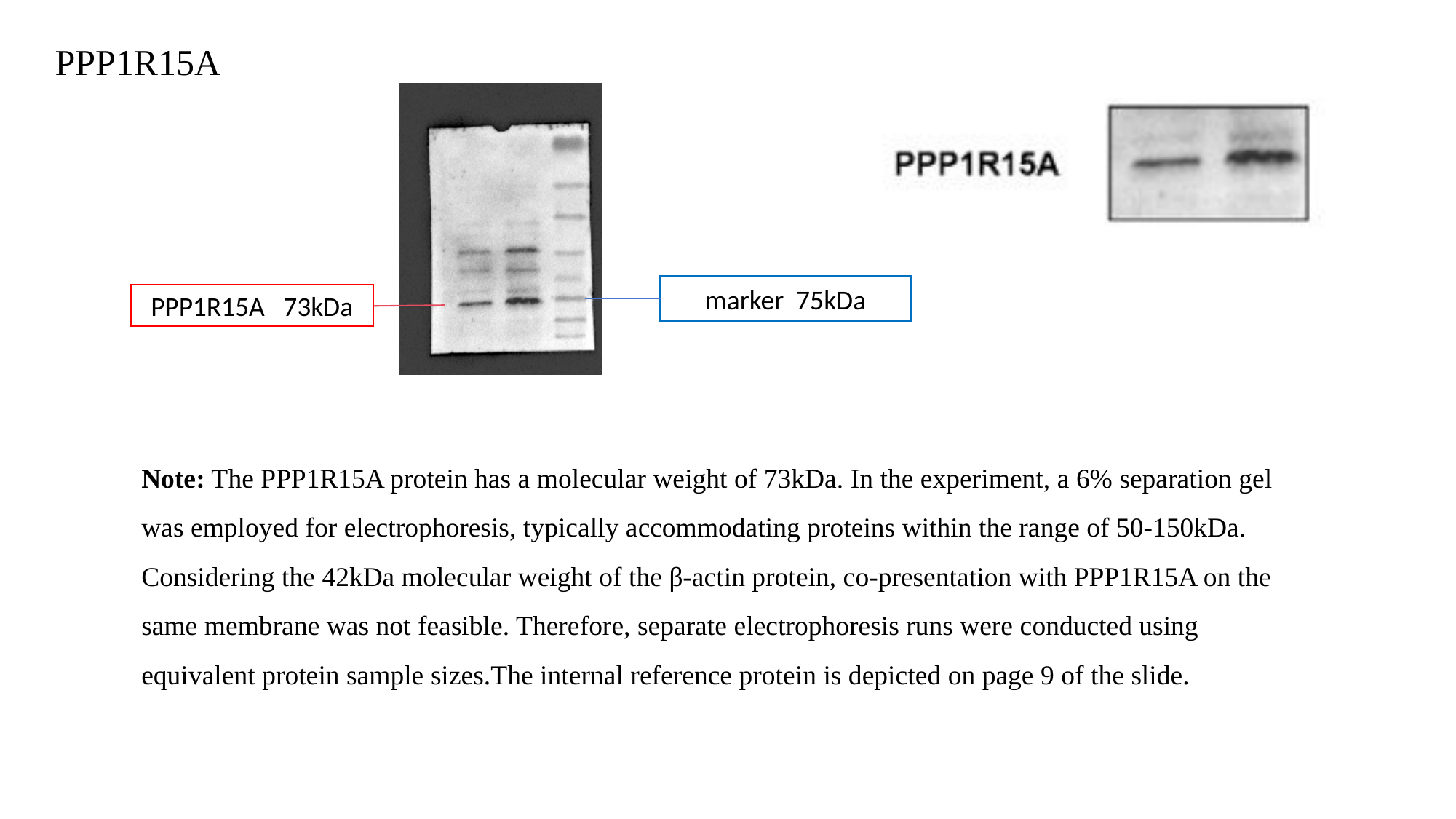

PPP1R15A
marker 75kDa
PPP1R15A 73kDa
Note: The PPP1R15A protein has a molecular weight of 73kDa. In the experiment, a 6% separation gel was employed for electrophoresis, typically accommodating proteins within the range of 50-150kDa. Considering the 42kDa molecular weight of the β-actin protein, co-presentation with PPP1R15A on the same membrane was not feasible. Therefore, separate electrophoresis runs were conducted using equivalent protein sample sizes.The internal reference protein is depicted on page 9 of the slide.

## Slide 6
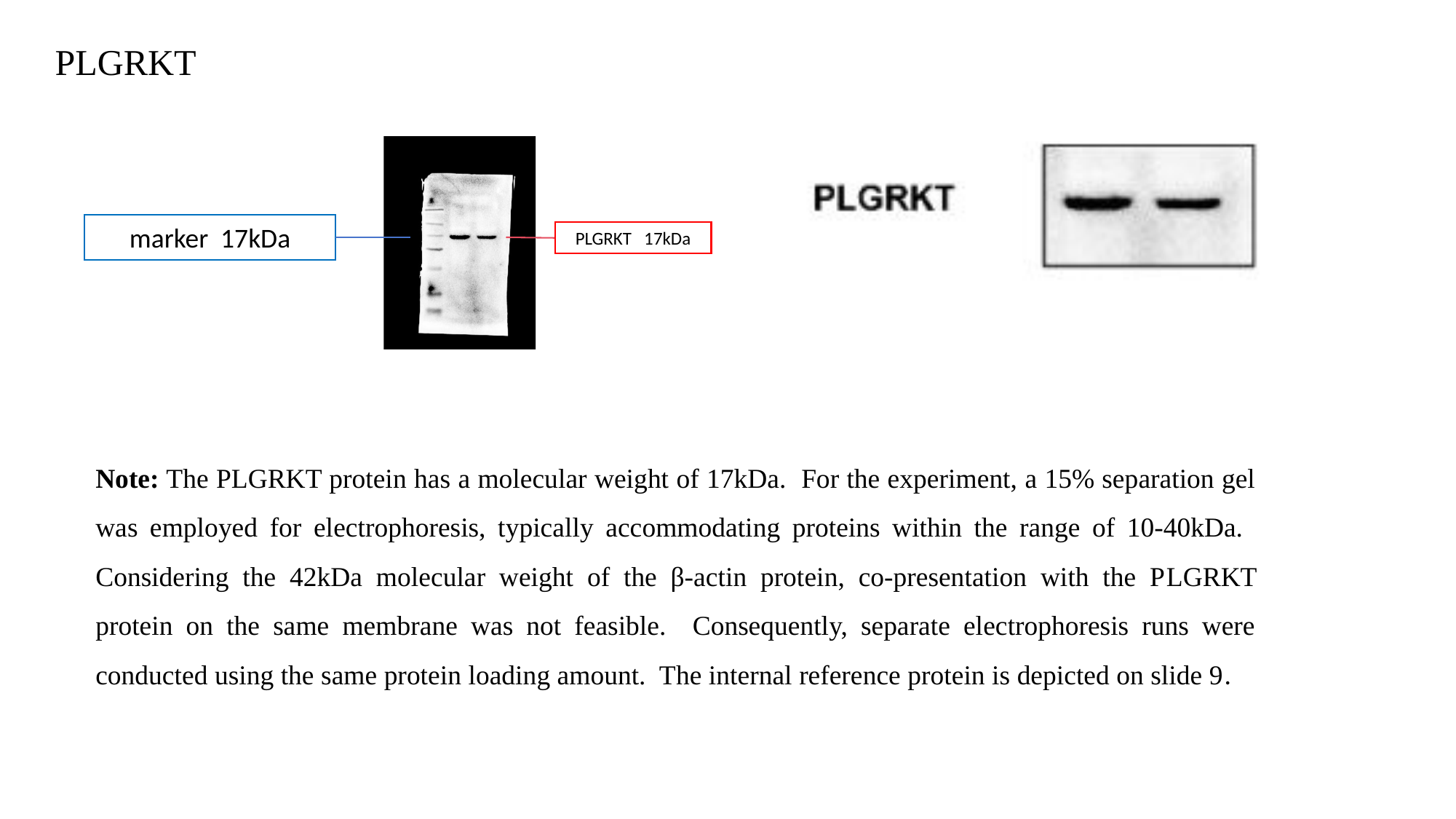

PLGRKT
marker 17kDa
PLGRKT 17kDa
Note: The PLGRKT protein has a molecular weight of 17kDa. For the experiment, a 15% separation gel was employed for electrophoresis, typically accommodating proteins within the range of 10-40kDa. Considering the 42kDa molecular weight of the β-actin protein, co-presentation with the PLGRKT protein on the same membrane was not feasible. Consequently, separate electrophoresis runs were conducted using the same protein loading amount. The internal reference protein is depicted on slide 9.

## Slide 7
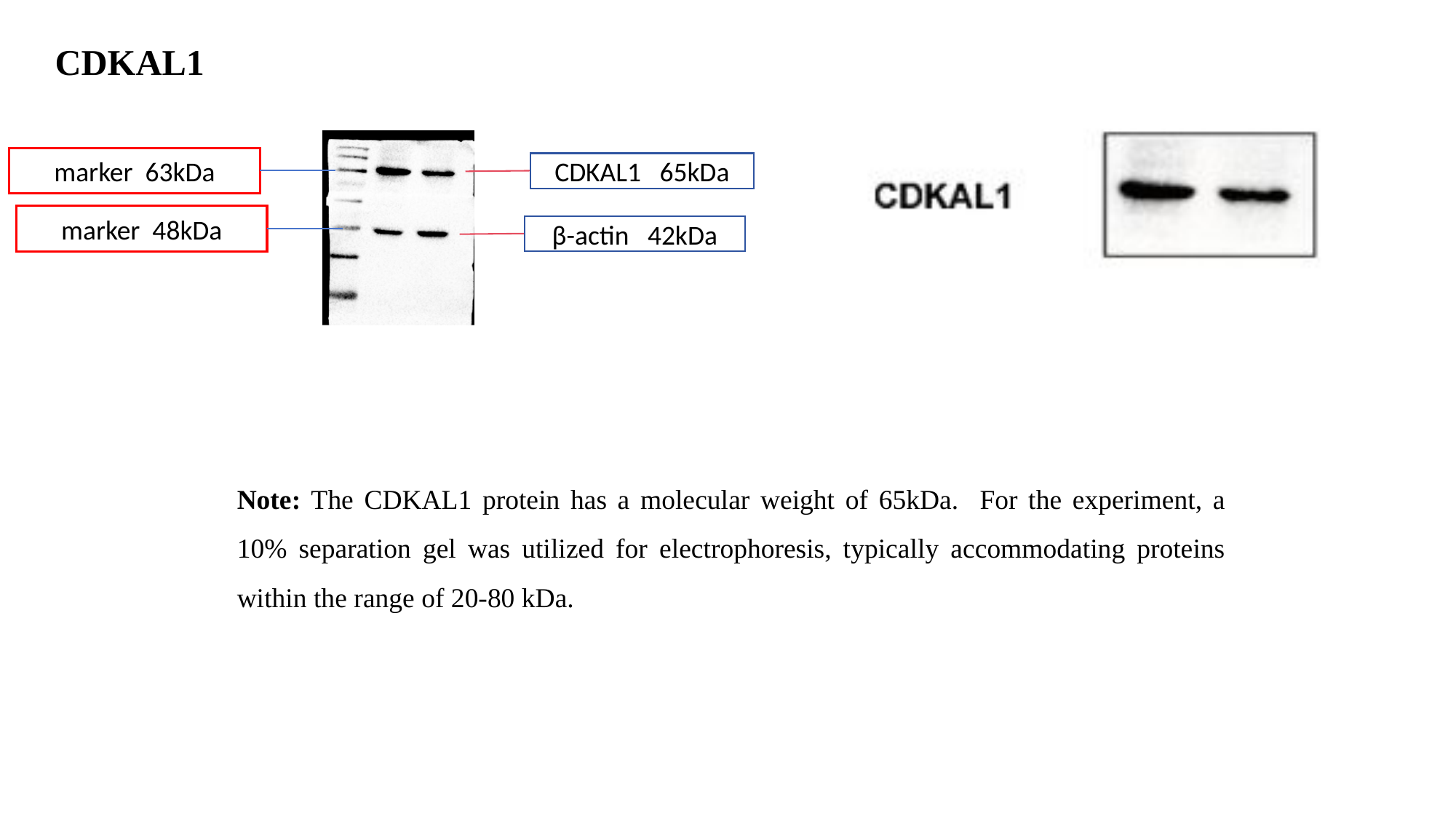

CDKAL1
CDKAL1 65kDa
β-actin 42kDa
marker 63kDa
marker 48kDa
Note: The CDKAL1 protein has a molecular weight of 65kDa. For the experiment, a 10% separation gel was utilized for electrophoresis, typically accommodating proteins within the range of 20-80 kDa.

## Slide 8
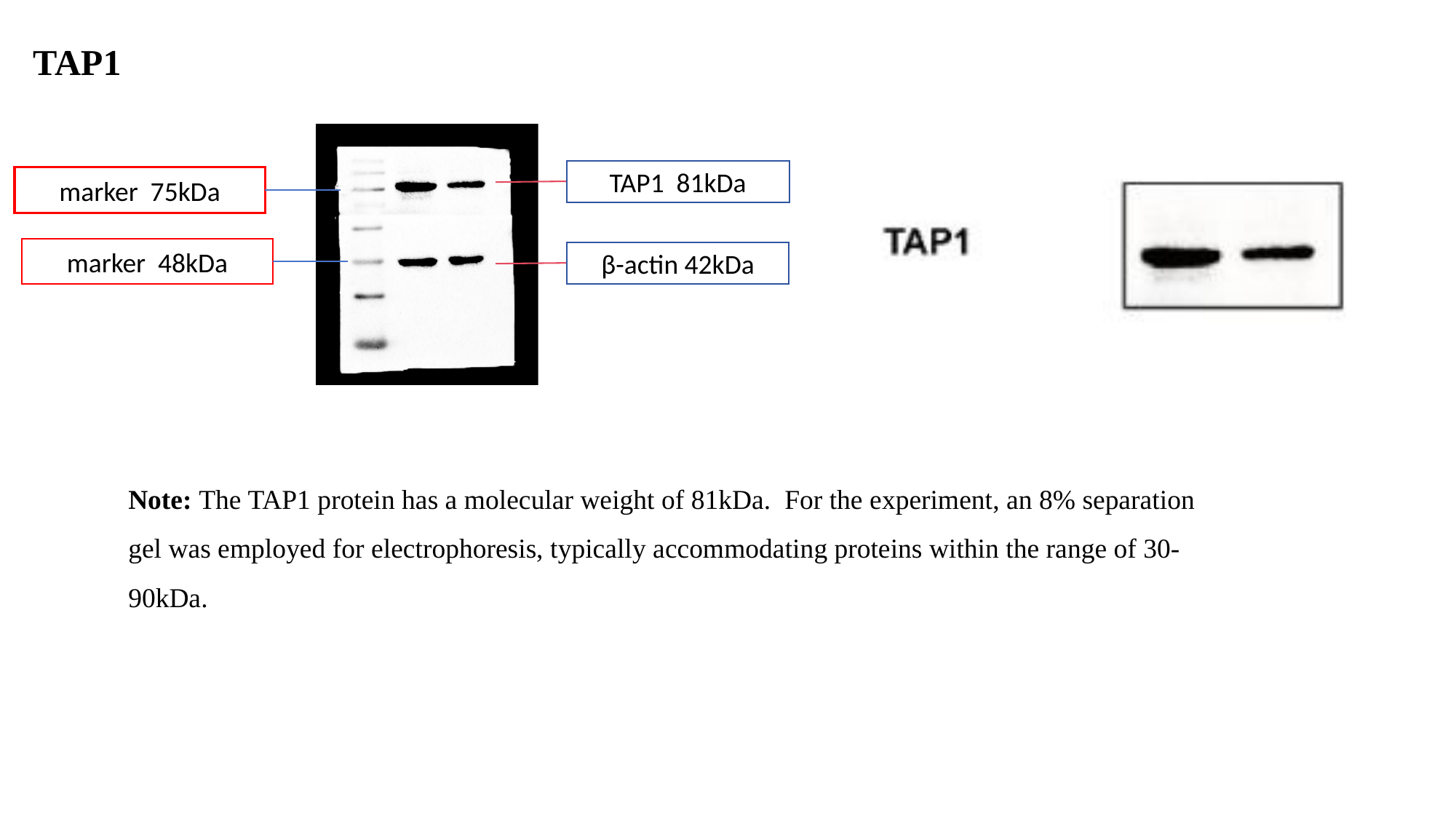

TAP1
TAP1 81kDa
β-actin 42kDa
marker 75kDa
marker 48kDa
Note: The TAP1 protein has a molecular weight of 81kDa. For the experiment, an 8% separation gel was employed for electrophoresis, typically accommodating proteins within the range of 30-90kDa.

## Slide 9
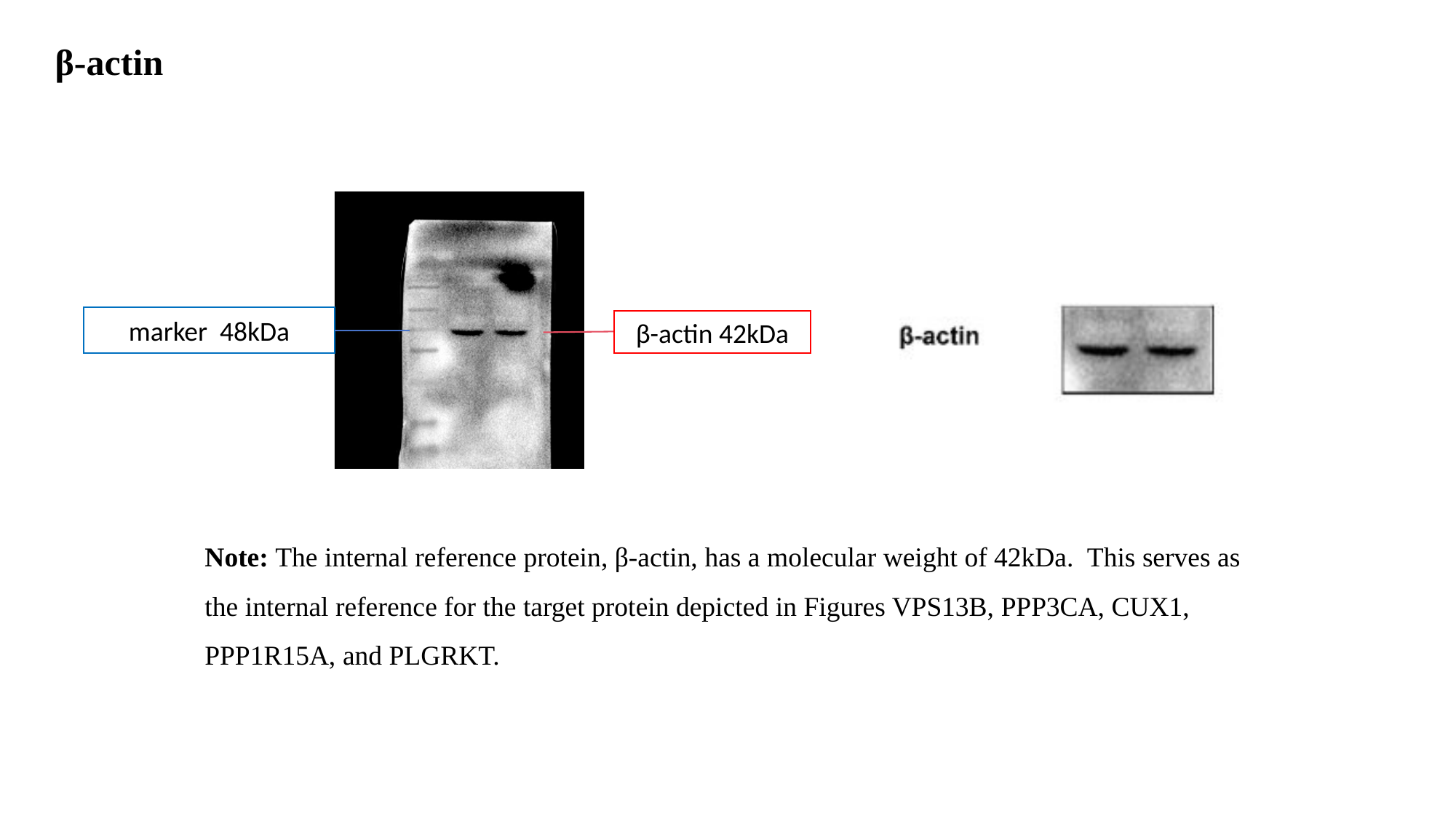

β-actin
β-actin 42kDa
marker 48kDa
Note: The internal reference protein, β-actin, has a molecular weight of 42kDa. This serves as the internal reference for the target protein depicted in Figures VPS13B, PPP3CA, CUX1, PPP1R15A, and PLGRKT.
